# YOLOv8s-Longan: a lightweight detection method for the longan fruit-picking UAV

**DOI:** 10.3389/fpls.2024.1518294

**Published:** 2025-01-22

**Authors:** Jun Li, Kaixuan Wu, Meiqi Zhang, Hengxu Chen, Hengyi Lin, Yuju Mai, Linlin Shi

**Affiliations:** ^1^ College of Engineering, South China Agricultural University, Guangzhou, China; ^2^ Guangdong Laboratory for Lingnan Modern Agriculture, Guangzhou, China; ^3^ State Key Laboratory of Agricultural Equipment Technology, Beijing, China

**Keywords:** longan, lightweight network, attention mechanism, YOLOv8-Longan network, target detection

## Abstract

**Introduction:**

Due to the limited computing power and fast flight speed of the picking of unmanned aerial vehicles (UAVs), it is important to design a quick and accurate detecting algorithm to obtain the fruit position.

**Methods:**

This paper proposes a lightweight deep learning algorithm, named YOLOv8s-Longan, to improve the detection accuracy and reduce the number of model parameters for fruitpicking UAVs. To make the network lightweight and improve its generalization performance, the Average and Max pooling attention (AMA) attention module is designed and integrated into the DenseAMA and C2f-Faster-AMA modules on the proposed backbone network. To improve the detection accuracy, a crossstage local network structure VOVGSCSPC module is designed, which can help the model better understand the information of the image through multiscale feature fusion and improve the perception and expression ability of the model. Meanwhile, the novel Inner-SIoU loss function is proposed as the loss function of the target bounding box.

**Results and discussion:**

The experimental results show that the proposed algorithm has good detection ability for densely distributed and mutually occluded longan string fruit under complex backgrounds with a mAP@0.5 of 84.3%. Compared with other YOLOv8 models, the improved model of mAP@0.5 improves by 3.9% and reduces the number of parameters by 20.3%. It satisfies the high accuracy and fast detection requirements for fruit detection in fruit-picking UAV scenarios.

## 1 Introduction

Longan, a special fruit native to tropical and subtropical regions, is favored for its unique flavor and rich nutrition. However, longan has a relatively short ripening period, and timely picking is essential to ensure fruit quality. At present, longan is mainly harvested manually. However, the manual picking of tall longan trees has high labor intensity and high operation risk. Therefore, developing agricultural robots that can automatically pick longan has great economic value. Although some researchers have developed fruit harvesting robots (Yang et al., 2023, it is necessary to develop more adaptive harvesting robots according to the growth characteristics of large longan trees to improve picking efficiency and reduce labor costs to promote the development of modern agriculture.

Robotic picking is currently being studied by a wide range of scholars (Shi et al., 2023; Dairath et al., 2023). He et al. built a robotic vision servo system for tomato picking utilizing a depth camera and a six-degree-of-freedom manipulator. The system utilizes depth and color information of fruit targets and adopts a coordinated control strategy for the hand and eye at different distances (He et al., 2021). Liang et al. developed a facility-based cultivation grape-picking robot using a monocular camera and a distance-measuring sensor to identify clusters and locate the fruit branch cutting points for fast, efficient, and low-loss grape picking (Liang and Wang, 2023). However, robotic arm-type picking devices suffer from limited operating range, low picking flexibility, and poor maneuverability, which limit the advantages of automated picking. Aiming at the string fruit growth characteristics of tall longan trees, further development of more adapted harvesting robots is needed to improve picking efficiency and reduce labor costs.

Compared with traditional ground-based mechanical equipment, the unmanned aerial vehicle (UAV) has a wide range of application prospects in fruit-picking tasks due to their smaller size, good maneuverability, and strong adaptability to complex terrain (Chen et al., 2024a; Lu et al., 2024; Zhaosheng et al., 2022). Longan fruit in the fruit tree shows the characteristics of irregular, inconspicuous, and widely distributed string fruit growth characteristics, and its natural background is more complex, prone to multiple clusters of longan string fruits overlapping each other, as well as by the fruit tree branches and leaves cover and so on. In order to achieve accurate detection of longan string fruits, deep learning target detection techniques have been applied to string fruit detection in agricultural work scenarios due to their ability to extract complex patterns and regularities by learning a large amount of data (Li et al., 2021; Ding et al., 2024, 2022). Among them, Li et al. proposed an improved YOLOv7-litchi detection algorithm by integrating ELAN-L and ELAN-A modules based on lightweight ELAN on the backbone network, which makes the network structure lightweight and provides a theoretical basis for mechanized lychee harvesting (Li et al., 2024). Huang et al. proposed Triplet-Large Kernel Attention (TLKA). The TLKA module inherits the advantages of channel attention and large kernel attention, and TLKA-YOLOv7 outperforms all other research models in grape string detection and segmentation and obtains more competitive results in yield prediction (Huang and Li, 2023). Chen et al. proposed an improved YOLOv7-based multi-task deep convolutional neural network (DCNN) detection model MTD-YOLOv7 with two additional decoders for detecting tomato fruit cluster ripeness based on YOLOv7 (Chen et al., 2024b). Liu et al. (2024) proposed the MAE-YOLOv8 model using YOLOv8s-p2 as the baseline and introduced MPDIoU as the regression loss function to accurately detect Qing crisp plum in the actual complex orchard environment. Meanwhile, YOLOv8 is compared with other YOLO series models. In the Backbone network part, the YOLOv8 model uses the DarkNet-53 network structure, uses C2f to replace the C3 module, and uses the faster SPPF module. In the Neck network part, the YOLOv8 model uses the PAN-FPN network structure that removes the convolution structure in the upsampling stage. In the Head network part, YOLOv8 uses the Decoupled-Head network structure to separate the classification and detection heads. The YOLOv8 model is an anchor-free model, which directly predicts the center of the object rather than the offset of the known Anchor box. These improvements make YOLOv8 show higher performance and accuracy in object detection tasks, which are more widely studied by scholars (Sun, 2024; Jiang et al., 2023; Wang et al., 2024).

The above research is dedicated to optimizing deep learning models to improve their ability to detect string fruits. However, in the practical problems of agricultural automated picking tasks, when the fruit-picking UAV performs the longan-picking task, limited by the endurance, computing resources, and dynamic characteristics of fast flight, a lightweight and high-precision object detection model is needed.

In response to the above challenges, the key issues addressed in this paper are mainly divided into two aspects: i) model lightweight and ii) recognition and detection accuracy improvement. Specifically, the model is lightweight to solve the problem of the limited endurance of UAVs. The high demand for complex neural networks for computing resources will increase energy consumption and affect the operation time and identification and detection efficiency of UAVs. The improvement of detection accuracy is to ensure that the UAV can accurately identify and locate the target fruit in the process of rapid flight, reduce the recognition error, and improve the picking accuracy. Due to the irregular, inapparent, and widely distributed characteristics of longan bunches on the fruit tree, traditional detection methods often have difficulty balancing between real-time performance and accuracy.

To this end, the YOLOv8s-Longan model is proposed in this paper. In this paper, we propose a novel solution to realize longan picking using the fruit-picking UAV. It will help to improve object detection accuracy for the vision-based fruit-picking UAV in natural environments. A dataset of UAV-collected longan images is built to train and evaluate object detection models. The main contributions of this paper are listed in the following three parts.

1. Considering the limited computing power and fast flight speed of the UAV, this paper first proposes a lightweight deep learning model, named YOLOv8s-Longan, to obtain real-time fruit location in complex backgrounds.
2. For model lightweight, the Average and Max pooling attention (AMA) attention module is designed and integrated into the DenseAMA and C2f-Faster-AMA modules on the proposed backbone network to reduce the number of parameters and the number of calculations to make the network lightweight.
3. For detection accuracy, a novel Inner-SIoU loss function is designed, and the cross-stage local network structure VOVGSCSPC module is integrated into the neck network, which improves the model’s ability to accurately locate the target longan and facilitates the UAV to move more stably to the designated location for picking.
4. The proposed model is actually developed on the UAV and occupies 18.1 MB of storage memory, which can process 45 to 50 images per second, and the average recognition accuracy of the real longan orchard scenario is 87.5%. It can meet the lightweight and accurate recognition of the longan fruits by the fruit-picking UAV.

## 2 Materials

### 2.1 Image acquisition equipment

**Figure 1:**
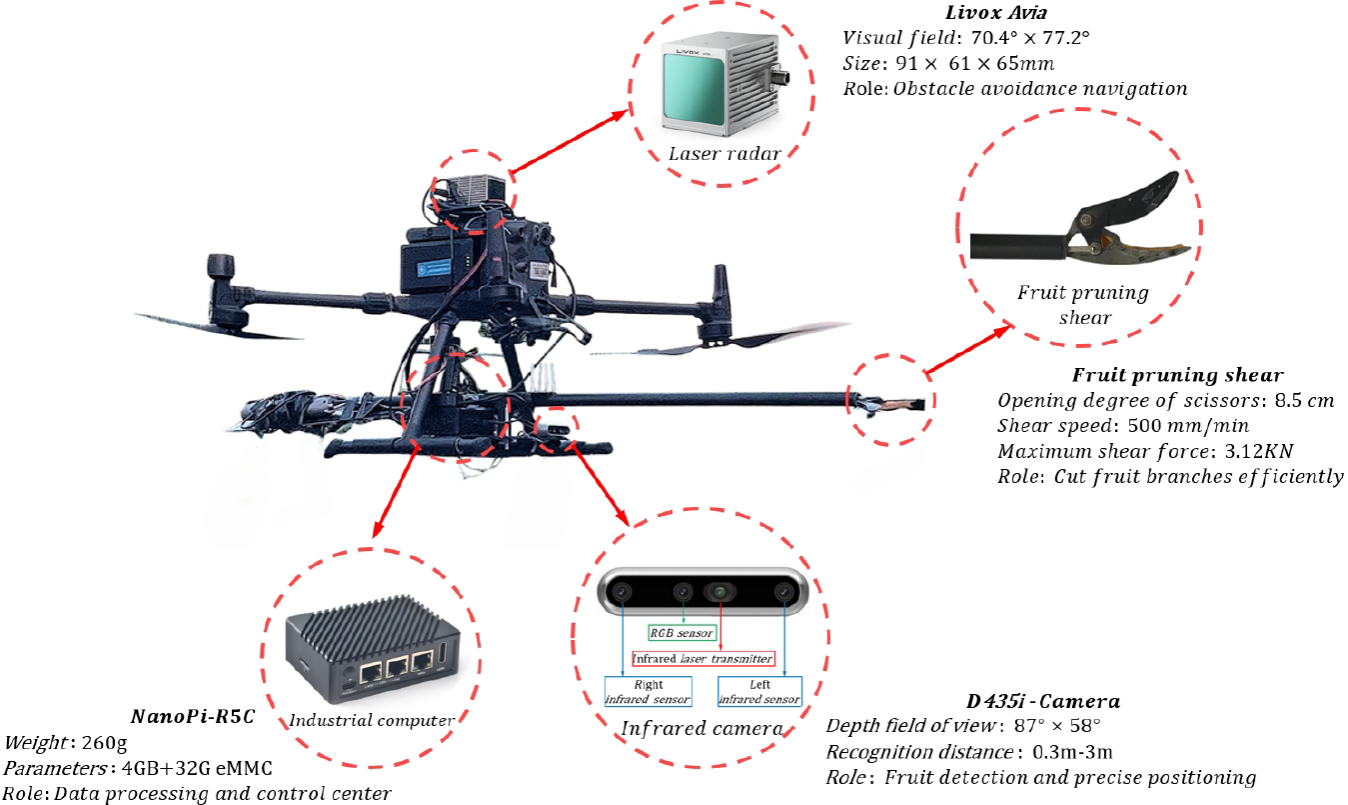
The structure of the fruit-picking UAV. UAV, unmanned aerial vehicle.

In this paper, the structure of the independently developed and designed fruit-picking UAV is shown in **Figure 1**. An RGB-D camera named RealSense D435i is installed for image acquisition, which combines the features of a color camera and an infrared camera. To enhance the model’s generalization, the images of Shek Kip and Chuliang longan are collected. To fully restore the real scene of UAV picking longan and the complexity of the orchard environment, the images within the range of 400–700 mm (near view) and 700–1,100 mm (far view) from the longan string are selected as the dataset. The dataset includes images taken under various lighting conditions, such as full sun and backlight, to ensure the acquired images are not disturbed by artificial shadows or lights.

### 2.2 Image dataset preprocessing

#### 2.2.1 Image filtering

The 1,070 collected images were screened and reviewed, and images with high-definition and rich details were selected. Images with poor quality, severe exposure, and only a single string of fruit were eliminated to ensure the accuracy and stability of the subsequent algorithm.

#### 2.2.2 Image flipping and brightness adjustment

Self-programmed image left–right flip and brightness adjustment algorithms are used to expand the image data to ensure the diversity of image data. In this way, the dataset is expanded from the original 438 longan images to obtain 2,460 images, and **Table 1** shows the statistics of the categories and numbers of images in the dataset.

**Table 1:** Image categories and number.

Influence factor	Image category	Number of images
Original image	/	438
Flip degree	Left and right flip	438
Light conditions	Very highlights (flag = 0.3:0.4) Highlights (flag = 0.5:0.8) Shadows (flag = 1.2:1.5) Polar shadows (flag = 2.5:3.0)	1,584

#### 2.2.3 Image annotation

For 2,460 images, manual annotation and classification label definition are performed, where string fruit means a string of longan from the first to the last branch on the fruiting mother branch. The annotated dataset is divided according to the ratio of the training set to the test set (4:1), and 1,968 training images and 492 test images are obtained. As shown in **Table 2**, the number of images and annotation information contained in the dataset are counted, and the images of the test set are grouped according to the set standards to prepare for the grouping test of the network model and to examine the effectiveness of the network model in various interference cases.

**Table 2:** Details of the dataset.

	Number of images	Number of bounding boxes
Total dataset	2,460	34,302
Train dataset	1,968	28,554
Test dataset	492	5,748

## 3 The YOLOv8s-Longan detection method

### 3.1 Overall network structure

**Figure 2:**
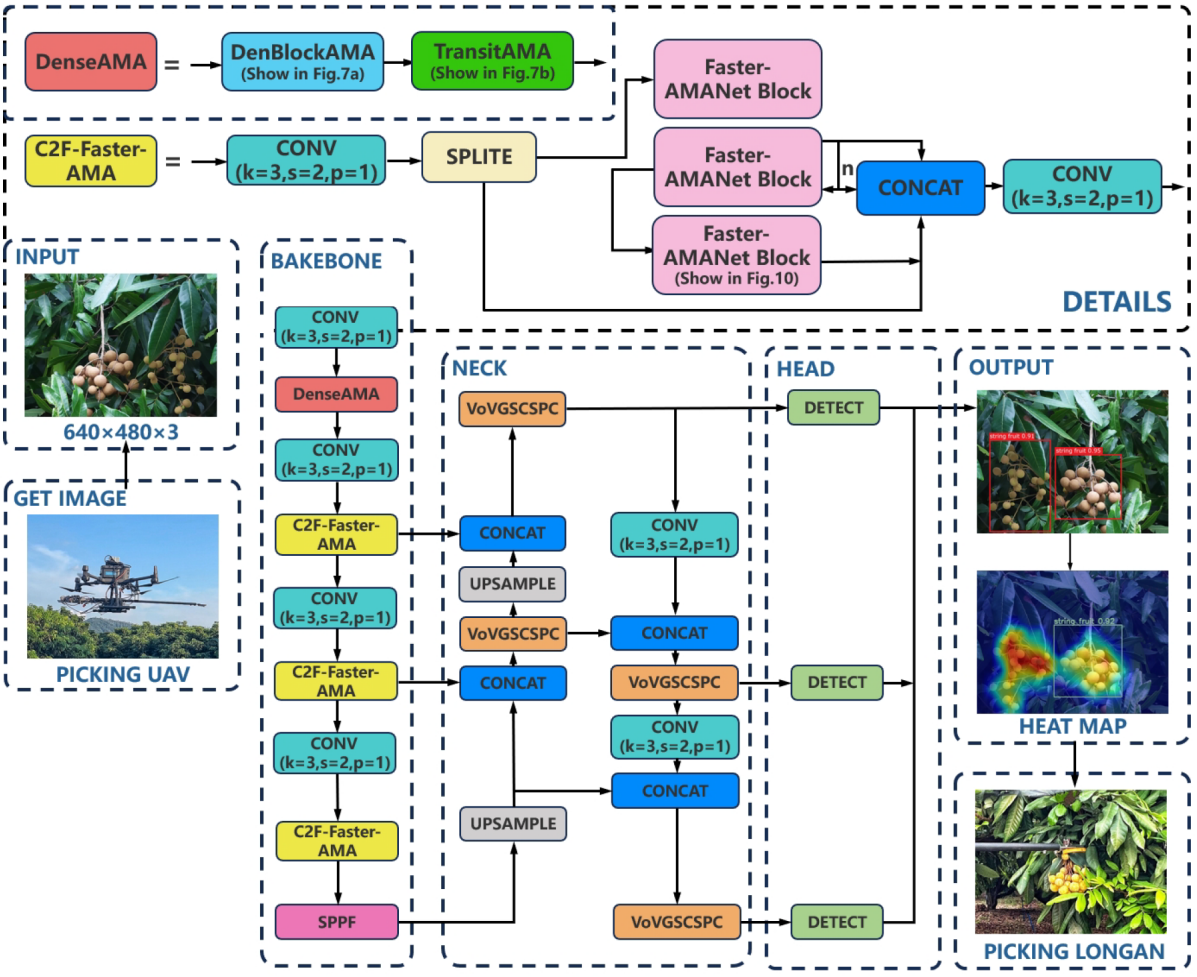
YOLOv8s-Longan network structure.

To improve the performance of the deep learning visual model for longan string fruit picking, the algorithm in this study is based on the YOLOv8 detection model to construct a lightweight YOLOv8s-Longan model, which is composed of three main parts: the backbone, neck, and head. The overall structure is shown in **Figure 2**. The detailed procedure of YOLOv8s-Longan is shown in **Algorithm 1**. The backbone serves as the backbone network of the model, consisting of the DenseAMA module, C2f-Faster-AMA module, and SPPF module. The input image is first passed through the densely connected DenseAMA module as the feature extractor to replace the first Conv and C2f combination module in the backbone network, which strengthens the feature learning ability of longan string fruit and mitigates the problem of insufficient features of longan string fruit in complex orchard environments. Then, the C2f-Faster-AMA module is used to replace the C2f module in the subsequent backbone network, which can significantly reduce the amount of computation and memory access, thus lightening the backbone network and improving the inference speed of the model, which is conducive to the real-time detection of longan string fruit by the UAV during flight. Finally, the multiscale features are fused through the SPPF module of the backbone network. The features of the same longan string fruit feature map at different scales are fused to enrich the semantic features of the longan string fruit feature map, improve the attention given to important details of the string fruit features, and enhance the quality of the features obtained by the model.

Algorithm 1 **Lightweight detection method of YOLOv8s-Longan.**

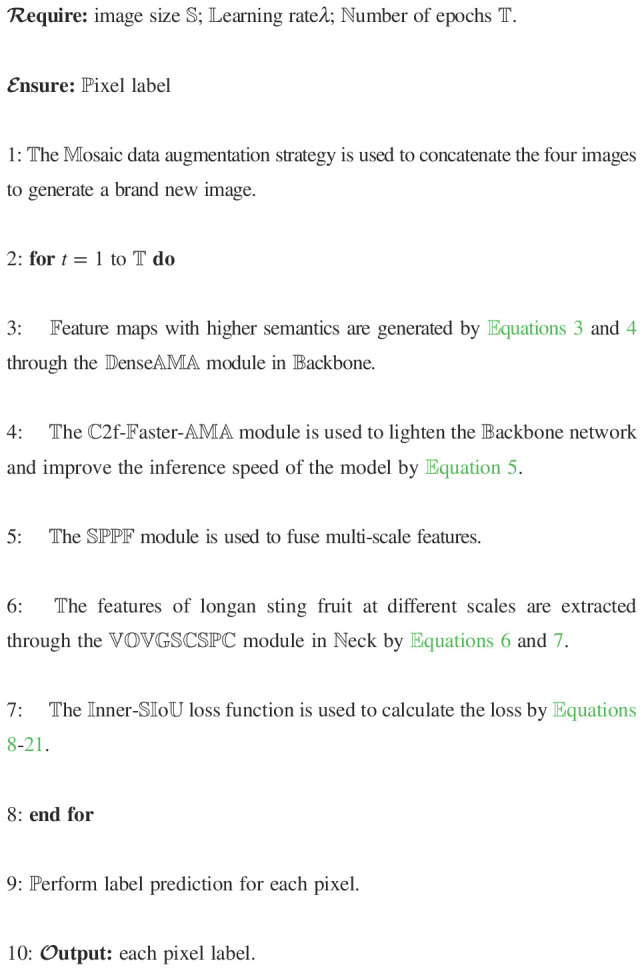

The neck is used as the pyramid multiscale feature fusion structure of the model, and the different scales of longan string fruit feature maps output by the backbone are fused to different degrees. The cross-stage local network structure of the VOVGSCSPC module is designed through the aggregation method to replace the C2f module in the neck to reduce the complexity of its network structure and make the YOLOv8s-Longan model easy to deploy to the terminal equipment of the fruit-picking UAV.

The head is used as the model detection output, and the bounding box is generated for the longan string fruit feature maps of different scales output by the neck. The Inner-SIoU loss function is used as the loss function of the target bounding box to improve the positioning ability and prediction accuracy of the target box so that the UAV can more accurately and quickly detect the position information of the longan string in the process of flight.

### 3.2 Improvement of the backbone network

#### 3.2.1 Proposed the AMA attention module

Longan fruits usually grow in the form of string fruits and show irregular, inconspicuous, and widely distributed features on the fruit trees. Moreover, the natural background of longan string fruits is complicated, and multiple clusters of longan string fruits overlap with each other, as well as being shaded by the branches and leaves of the fruit trees.

Meanwhile, different levels of longan feature maps usually have different background noise distributions and also generate redundant information due to differences in scale and location of longan string fruit feature maps. Therefore, in this paper, feature fusion is used to suppress the background noise of individual longan string fruit feature maps and generate more discriminative feature representations. In order to suppress the interference of negative information such as multiple cluster occlusion of longan string fruits and occlusion of fruit tree branches and leaves, the authors propose an AMA module, which is weighted by average pooling and maximum pooling, to reduce the negative impact of redundant information and noise on the network, improve the network’s attention to longan string fruits, and help the model to focus on the most distinguishable and important features in the input.

**Figure 3:**
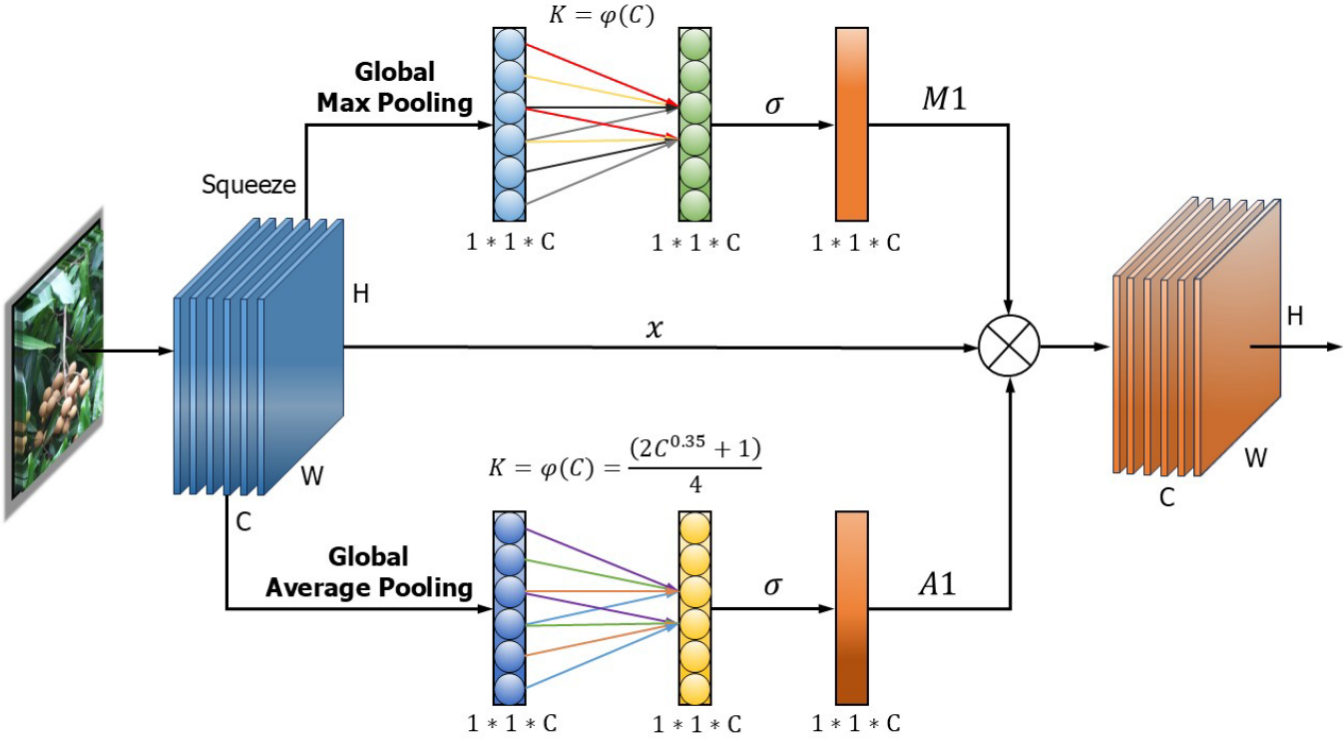
Structure of the AMA attention module. AMA, Average and Max pooling attention.

The structure of the AMA attention module is shown in **Figure 3**. First, one-dimensional convolution is used to replace the fully connected layer, effectively reducing the weight parameters and increasing the inference speed, where *W*, *H*, and *C* are the width, height, and channel size of the feature vector, respectively. Then, global average pooling (GAP) and global maximum pooling (GMP) are performed on the last convolution output to aggregate the convolution features without dimensionality reduction.

Subsequently, channel feature learning is performed with the same dimension, and one-dimensional convolution is used to quickly capture the cross-channel information interaction between each channel and its nearly *K* adjacent channels. Thus, there is a non-linear mapping between *K* and *C*, as in Equations 1 and 2.


(1) $$C=\omega \left(K\right)=\left|{\left(\frac{gK-b}{a}\right)}^{\frac{1}{0.35}}\right|,$$

(2) $$K=\varphi \left(C\right)=\left|\left(\frac{aC^{0.35}+b}{g}\right)\right|,$$

where *a* = 2, *b* = 1, and *g* = 4.

The activation value of the one-dimensional convolution is calculated by the sigmoid activation function, and different weights are obtained to show the relevance and importance of the longan string features between channels. Finally, the learnable weight coefficients (A1, M1) of each channel are generated by GAP and GMP. Then, the weight of each channel is weighted to the original input feature map to complete the recoding of each channel feature so that the important features are assigned large weights to be enhanced, and the effective longan string fruit features are enhanced. Instead, the negative environmental features of ineffective nature are assigned a small weight to suppress.

The AMA attention module avoids information loss caused by mapping the input longan features to low dimensions. Additionally, it can capture cross-channel interactions effectively, better capture the important feature information of the target to be detected, enhance the feature extraction ability of the network, and make the model use global features to distinguish the image information level. In addition, this AMA attention module has fewer parameter requirements, which avoids the excessive complexity of the model and compensates for the loss in accuracy caused by the model being lightweight, increasing the effectiveness of channel learning attention and leading to obvious performance gains in the network. It is beneficial to integrate into the subsequent DenseAMA and C2f-Faster-AMA modules more effectively and improve the module’s longan string fruit feature extraction ability.

#### 3.2.2 Proposed the DenseAMA module

In the detection of missed fruit in the agricultural field, the longan background image usually has the problems of unobvious features, complexity, and redundancy. Using the feature extraction module C2F developed based on natural view images may lead to insufficient extracted longan string fruit feature information, which limits the performance of the model in detection tasks. To this end, a densely connected DenseAMA module is proposed as a feature extractor to replace the first Conv and C2f combination module in the backbone network, which is used to extract features of various scales from the input image, and the output of each layer is directly connected with the input of all subsequent layers. This connection makes the information flow of the network more sufficient, helps to prevent the vanishing gradient problem, and can use low-level features to supplement high-level features.

**Figure 4:**
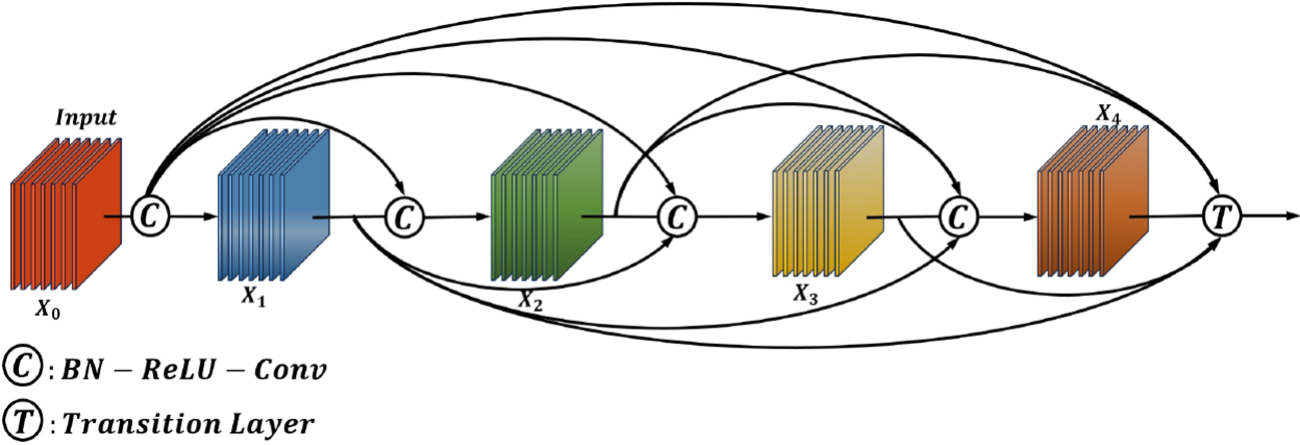
Schematic diagram of the DenseNet structure.


In computer vision, the main idea of DenseNet is to build dense connections, that is, to promote the reuse of features by connecting features between different channels (Jia et al., 2023; Cai et al., 2022). These properties allow DenseNet to maintain low model parameters and computational costs. The dense connection mechanism of DenseNet is shown in **Figure 4**, and its expression is below.

(3) $$X_{l}=W_{l}*\left[X_{0},X_{1},\ldots ,X_{n},\ldots ,X_{L-1}\right],$$

$X_{n}$ is the feature output of each layer through the convolutional network, $W_{l}$ is the weight of each dense layer, where *l* is the layer index, and ∗ is the composite function of operations such as batch normalization (BN), rectified linear unit (ReLU), pooling, or convolution.

DenseNet has multiple DenseBlocks, the inner layers of each DenseBlock are densely connected DenseLayer modules (by superposition rather than addition), and the dense blocks of different DenseBlocks are downsampled by transition layers. In this paper, the original DenseNet121 is used as the basic structure, and the H-swish activation function and the AMA attention mechanism are connected in the DenseBlock and transition layers to obtain the DenseAMA module.

Moreover, the H-swish (Sunkari et al., 2024; Mercioni and Holban, 2020) activation function has a low computational cost and comprises simple multiplication and addition operations, which can be calculated faster in model inference and training. The equation is shown in Equation 4:


(4) $$\begin{array}{ll} \text{HardSwish}\left(x\right) & =x\times \text{HardSigmiod}\left(x\right)\\ \; & =x\times \frac{\text{ReLu}6\left(x+3\right)}{6}\\ \; & =x\times \{\begin{array}{ll} 1, & x\leq 2\\ \frac{x}{6}+\frac{1}{2}, & -3\leq x\leq 3\\ 0, & x\leq 3 \end{array} \end{array}.$$

It shows that H-swish activation functions have strong similarities in terms of upper and lower boundaries, smoothness, and monotonicity. After replacing the sigmoid activation function with the H-swish activation function, the number of parameters and the calculations in the model can be effectively reduced. When the backpropagation algorithm is trained, the H-swish activation function has a lower gradient saturation problem, which means that it is easier to train in the deep neural network, which can effectively enhance the feature extraction ability and eliminate the potential accuracy loss. Therefore, the H-swish activation function is more suitable for improving mode performance.

**Figure 5:**
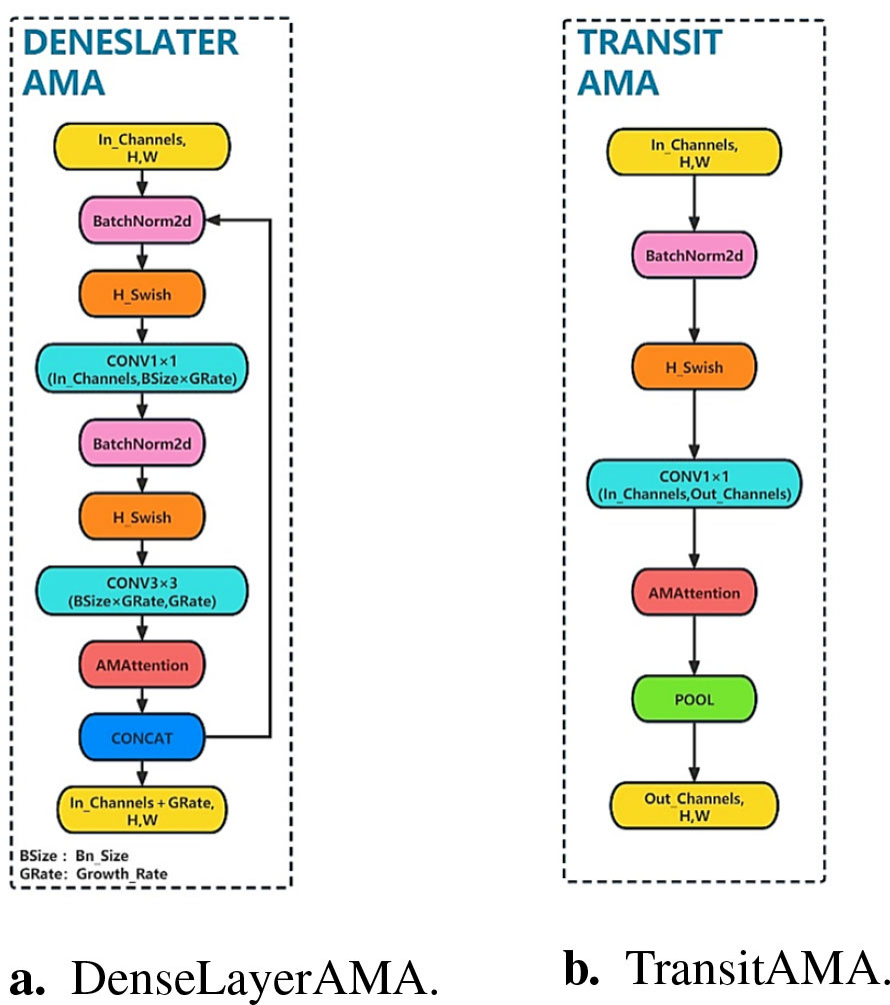
Local structure diagram of the DenseAMA module. **(A)** DenseLayerAMA. **(B)** TransitAMA.

The DenseAMA module consists of three stages, where the first and second stages form the DenseLayerAMA layer and the third stage forms the TransitAMA layer, as shown in **Figure 5**. In the first stage, a BN operation is performed on the input longan feature map, and the H-swish activation function is used to activate the feature map. Then, a 1 × 1 convolution kernel is used to reduce the number of parameters.

The second stage is similar to the first. The input feature map is batch normalized and activated by the H-swish activation function. Then, a 3 × 3 convolution kernel is used to convolve the feature map. Finally, to reduce the number of parameters and calculations in the model as much as possible, the AMA attention mechanism module designed in this paper is added after the 3 × 3 convolution operation in the second stage, and the AMA attention module is used to extract features of the string fruit feature map to enhance the utilization ability of longan string fruit features. The DenseLayerAMA layer structure is shown in **Figure 5A**.

The TransitAMA layer in the third stage first inputs the feature map for the BN operation and uses the H-swish activation function for activation. It is processed by the AMA attention mechanism, and the average pooling operation is performed on the processed feature map to reduce the size of the input feature map by half. It can better reduce the spatial dimension of the feature map and the number of calculations and increase the receptive field size to better capture global information. The transition layer of the third stage connects the two adjacent dense blocks of the first and second stages to each other, which reduces the size of the feature map and plays the role of a compression model. The network structure of the TransitAMA layer is shown in **Figure 5B**.

The DenseAMA module is used to replace the first Conv and C2f combination modules in the backbone network. The DenseAMA module can effectively take advantage of feature reuse while retaining the original string fruit feature information and significantly enhancing its semantics, making the low-level features richer and more detailed, and generating feature maps with higher semantics. This method helps to alleviate the problem that the longan string fruit features in agricultural scenes may be submerged by redundant background information when the depth of the model increases so that the UAV can accurately and effectively identify longan string fruit during flight and improve the adaptability to complex environments.

#### 3.2.3 Proposed C2f-Faster-AMA module

Although the accuracy of the YOLOv8 algorithm is improved compared with that of the previous version, the model is relatively complex and has a large number of parameters. When deploying the model in the field, the requirements for equipment performance are too high, and the model is not suitable for fruit-picking UAV terminal equipment. Therefore, the C2f module is improved to reduce the number of parameters and the model size, which overcomes the shortcomings of the YOLOv8 network in that the number of model parameters is too large and deployment is difficult.

Therefore, the simpler C2f-Faster-AMA module is proposed with the PConv convolution way to replace the last three C2f modules in the backbone network. By reducing the computations and memory access to extract features effectively, it can dynamically learn the relationships between different parts of the input, better understand the relationships and dependencies between longan data, and improve the performance of the YOLOv8s-Longan model.

**Figure 6:**
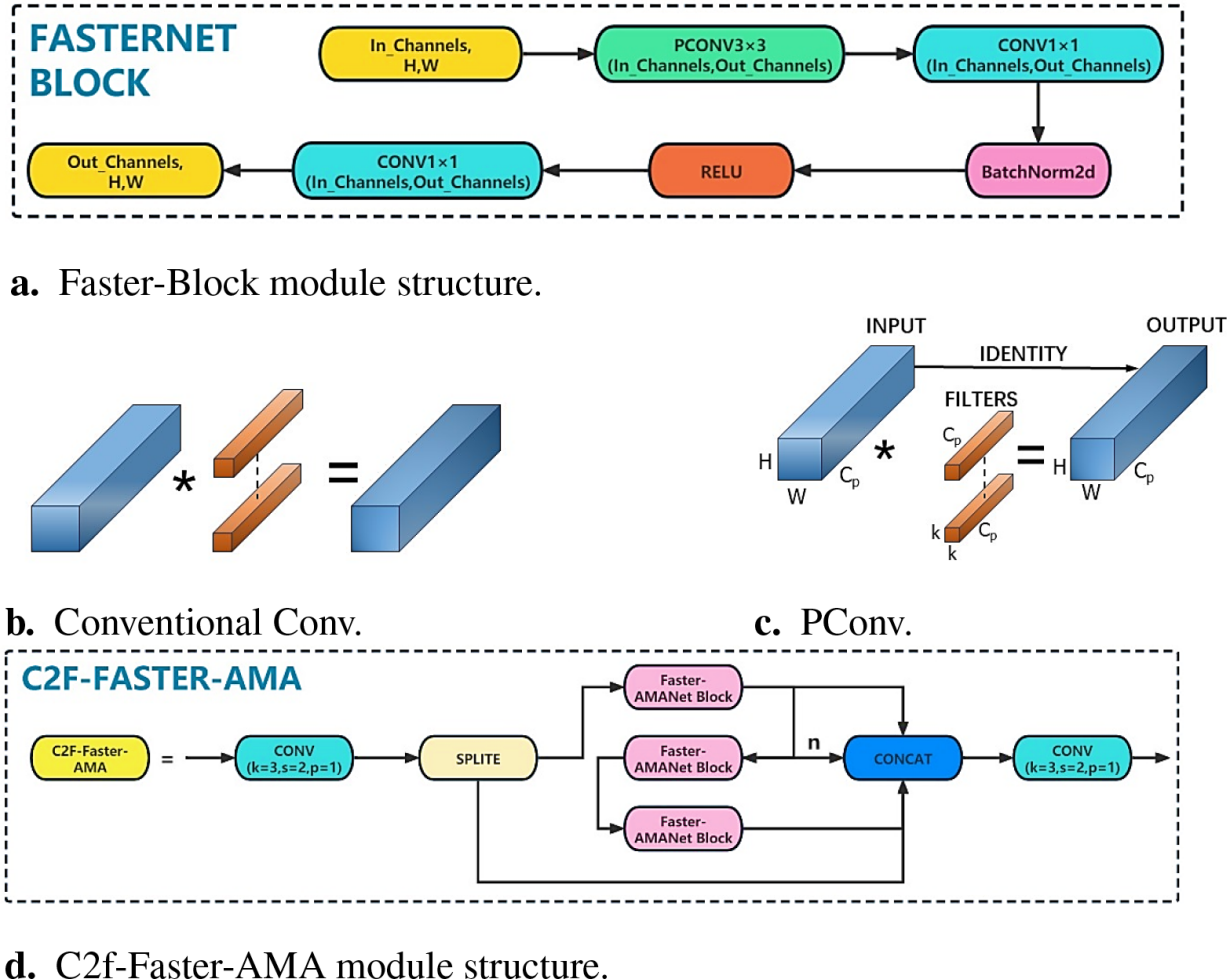
The specifics of the C2f-Faster-AMA module. **(A)** Faster-Block module structure. **(B)** Conventional Cony. **(C)** PCony. **(D)** C2f-Faster-AMA module structure.

Inspired by the FasterNet network, the bottleneck in the C2f module is replaced by Faster-Block, which reduces FLOPs while maintaining high FLOPS. The structure of the Faster-Block module is shown in **Figure 6A**.

Faster-Block consists of PConv and regular Conv modules. The PConv module can reduce both computational redundancy and memory access. The working principle of the PConv module is shown in **Figures 6B, C**. It shows that PConv only applies conventional Conv for spatial feature extraction on the part of the input channels, and the remaining channels remain unchanged. For consecutive or regular memory access, we compute the first or last consecutive *c_p_* channel as a representative of the entire feature map. Without loss of generality, the input and output feature maps have the same number of channels, as shown in Equation 5.


(5) $$\begin{array}{c} h\times w\times 2c_{p}+k^{2}\times c_{p}^{2}\approx h\times w\times 2c_{p} \end{array}$$

where *h*, *w*, and *c_p_* represent the height, width, and number of channels of the feature map, and *k* represents the size of the convolution kernel.

The Faster-AMANet block module is obtained by integrating the AMA attention mechanism into the FasterNet block and replacing the bottleneck in C2f to obtain the C2f-Faster-AMA module. The C2f-Faster-AMA module is used to replace the last three C2Fs in the backbone network, which can reduce the redundant calculation and memory access of the model, extract spatial features more effectively, and better understand the connections and dependencies between longan data. Thus, the lightweight and real-time detection of the YOLOv8s-Longan model is ensured, so the UAV can adjust its flight attitude according to the detection results of the vision model in real-time and realize safe and stable picking work. The C2f-Faster-AMA module structure is shown in **Figure 6D**.

### 3.3 Integration into the neck structure of VOVGSCSPC

By integrating the cross-stage local network structure of the VOVGSCSPC module designed by the fusion method, the C2f module in the neck part is replaced to fuse multiple longan string feature maps of different scales better (Xu et al., 2023; Zhu et al., 2024). The VOVGSCSPC module can extract richer semantic information, and multiscale feature fusion can help the model better understand the global and local information of the longan image and improve the perception and expression ability of the model.

**Figure 7:**
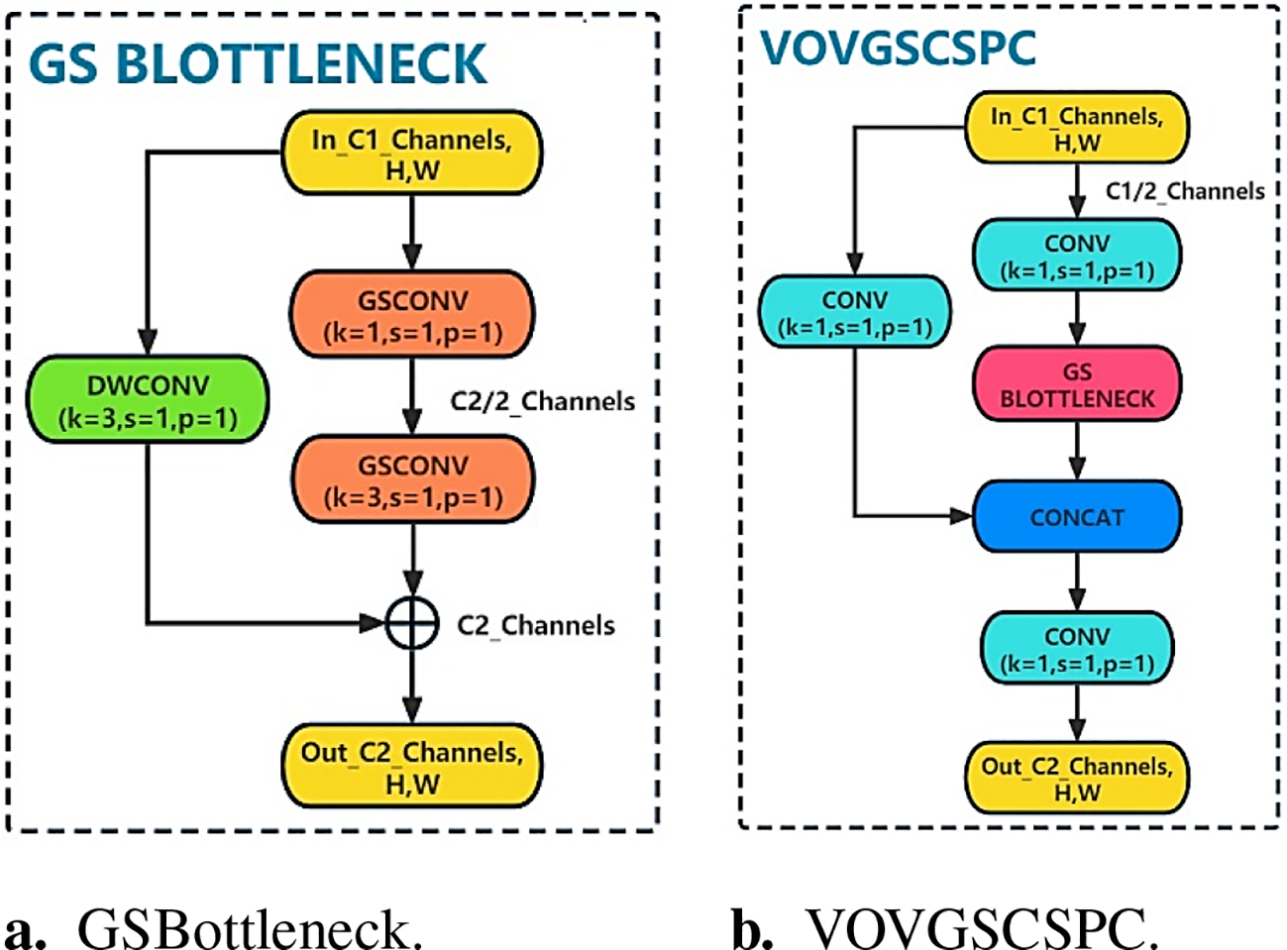
VOVGSCSP module structure. **(A)** GSBottleneck. **(B)** VOVGSCSPC.

To further reduce the model complexity, through the idea of ResNet, the VOVGSCSPC module is introduced to replace the original C2f module. The VOVGSCSPC module uses a cross-stage local network designed by the aggregation method, and the structure is shown in **Figure 7**. In GSBottleneck, the idea of a residual is adopted. The output is obtained by adding the residual of the input feature map after two GSConv convolutions and one DWConv depth convolution.

The above process is expressed as


(6) $$GSB_{out}=F_{GSC}\left(F_{GSC}\left(\alpha {\left(X_{C_{1}}\right)}_{\frac{C_{1}}{2}}\right)\right)+\alpha {\left(X\right)}_{\frac{C_{1}}{2}},$$

(7) $$VOVGSCSPC_{out}=\alpha \left(Concat\left(GSB_{out},\alpha \left(X_{C_{1}}\right)\right)\right),$$

where *C*_1_ is the number of channels of the input feature map $X_{C_{1}}$ , *α* is the conventional convolution, $GSB_{out}$ is the output of GSBottleneck, and $VOVGSCSPC_{out}\;$ is the final output of this module. The VOVGSCSPC neck structure balances the accuracy and speed of the model well and reduces the complexity of the calculation and network structure, making the YOLOv8s-Longan model lightweight and easier to deploy for fruit-picking UAV terminal equipment while maintaining sufficient accuracy and utilization of the extracted features.

### 3.4 Improvement of the Inner-SIoU loss function

The angle between the real bounding box and the predicted bounding box is ignored in different detection tasks to compensate for the existing IoU loss function, resulting in weak generalization ability and slow convergence speed in the training process, which easily results in a poor model. In this paper, the InnerSIoU loss function is proposed to capture the location information of defects more accurately and further improve the robustness of the algorithm.

In the Inner-SIoU, the use of an auxiliary bounding box is proposed to calculate the loss to accelerate the bounding box regression process, and the scale factor ratio is introduced to control the scale of the auxiliary bounding box. By using auxiliary bounding boxes of different scales for different datasets and detectors, we can overcome the limitations of existing methods in terms of their generalizability.

**Figure 8:**
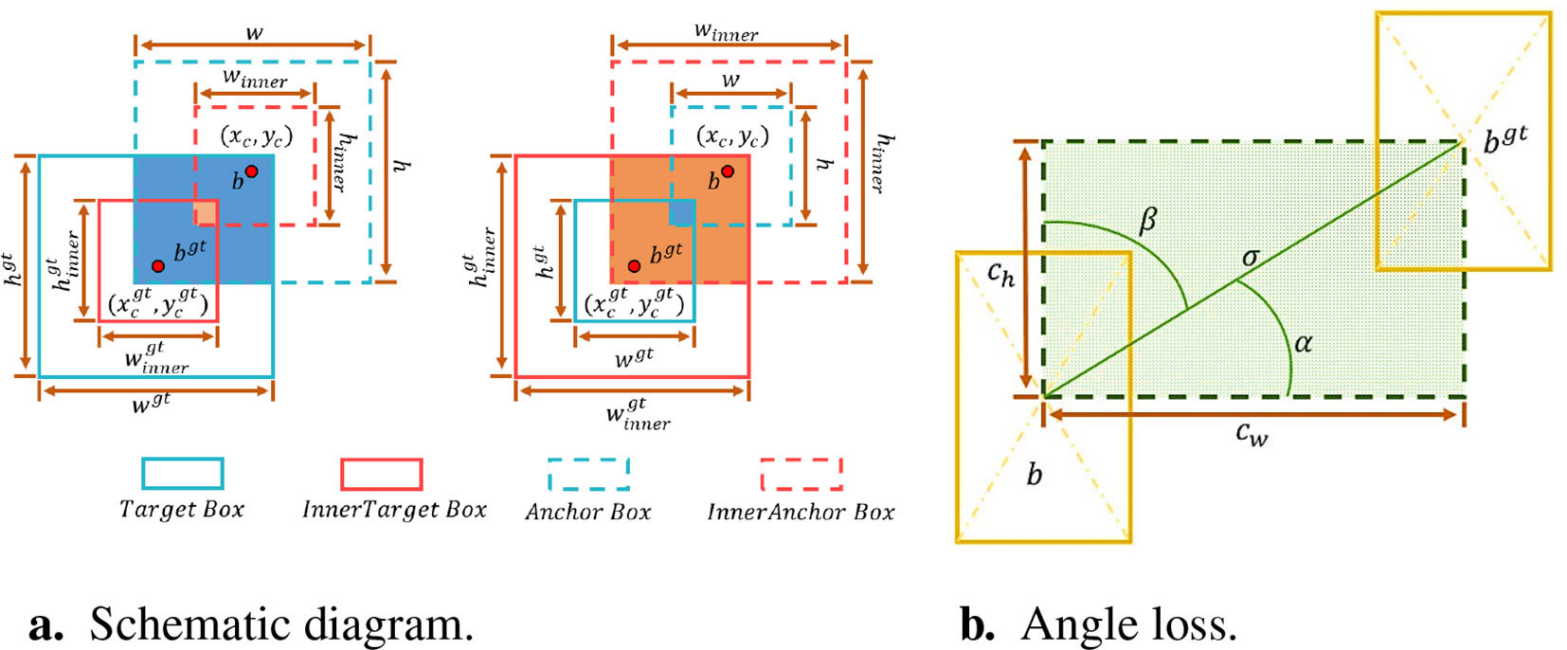
The specifics of the Inner-SIoU. **(A)** Schematic diagram. **(B)** Angle loss.


As shown in **Figure 8A**, the Ground truth and Anchor boxes are $b^{gt}$ and *b*, respectively. ( $x_{c}^{gt}$ , $y_{c}^{gt}$ ) is the center point of the GT box and the center point of the inner GT box, while the center point of the Anchor box and the inner Anchor box is denoted by (*x_c_*, *y_c_*). The width and height of the GT box are denoted by *w^gt^* and *h^gt^*, respectively, while the width and height of the Anchor box are denoted by *w* and *h*, respectively. The variable “ratio” corresponds to the scaling factor and is typically in the range [0.5, 1.5]. The relevant formulas are shown in Equations 8 and 14, which describe the adjustment process of the Anchor box with respect to the GT box. In these formulas, the Anchor box is scaled and displaced by the scaling factor ratio.

(8) $$b_{l}^{gt}=x_{c}^{gt}-\frac{\omega^{gt}\times ratio}{2},\;b_{r}^{gt}=x_{c}^{gt}+\frac{\omega^{gt}\times ratio}{2},$$

(9) $$b_{t}^{gt}=y_{c}^{gt}-\frac{h^{gt}\times ratio}{2},\;b_{b}^{gt}=y_{c}^{gt}+\frac{h^{gt}\times ratio}{2},$$

(10) $$b_{l}=x_{c}-\frac{\omega \times ratio}{2},\;b_{r}=x_{c}^{gt}+\frac{\omega \times ratio}{2},$$

(11) $$b_{t}=y_{c}-\frac{h\times ratio}{2},\;b_{t}=y_{c}^{gt}+\frac{h\times ratio}{2},$$

(12) $$\begin{array}{c} inter=\left(\text{min }\left(b_{r}^{gt},b_{r}\right)-\text{max }\left(b_{l}^{gt},b_{l}\right)\right)\\ \times \left(\text{min }\left(b_{b}^{gt},b_{b}\right)-\text{max }\left(b_{t}^{gt},b_{t}\right)\right), \end{array}$$

(13) $$union=\omega^{gt}\times h^{gt}\times ratio^{2}\;+\omega \times h\times ratio^{2}-inter,$$

(14) $$IoU^{inner}=\frac{inter}{union}.$$

The Inner-SIoU loss function inherits some characteristics of IoU and has unique characteristics. The range of Inner-SIoU and IoU loss functions is the same, which is [0, 1]. Since there is only a scale difference between the auxiliary bounding box and the actual bounding box, the loss function is calculated in the same way. Therefore, the Inner-IoU bias curve shows a similar trend to the IoU bias curve.

Additionally, the Inner-SIoU loss function redefines the loss index by the angle of the regression vector, which comprises three functions: angle loss, distance loss, and shape loss (Dong and Duoqian, 2023; Lawal et al., 2023). Here, the angle loss is defined as


(15) $$\text{Λ}=1-2{\text{sin}}^{2}\left(\text{arcsin }x-\frac{\pi }{4}\right),$$

(16) $$x=\frac{c_{h}}{\sigma }=\text{sin }\alpha ,$$

(17) $$\sigma =\sqrt{{\left(b_{c_{x}}^{gt}-b_{c_{x}}\right)}^{2}+{\left(b_{c_{y}}^{gt}-b_{c_{y}}\right)}^{2}},$$

(18) $$C_{h}=\text{max }\left\{b_{c_{y}}^{gt},b_{c_{y}}\right\}-\text{min }\left\{b_{c_{y}}^{gt},b_{c_{y}}\right\},$$

where $\left(b_{c_{x}}^{gt},\;b_{c_{y}}^{gt}\right)$ are the real bounding box coordinates, $\left(b_{c_{x}},\;b_{c_{y}}\right)$ are the predicted bounding box coordinates and *a* is the vector Angle. The angle loss is shown in **Figure 8B**.

The distance loss is defined as Equations 19 and 20:


(19) $$\text{Δ}=\underset{t=xy}{\Sigma }\left(1-{\text{exp}}^{-\gamma \rho_{t}}\right).$$

where


(20) $$\rho_{x}={\left(\frac{b_{cx}^{gt}-b_{cx}}{c_{w}}\right)}^{2},\rho_{y}={\left(\frac{b_{cy}^{gt}-b_{cy}}{c_{h}}\right)}^{2},\gamma =2-\text{Λ}$$

The shape loss is defined in Equations 21 and 22:


(21) $$\text{Ω}=\underset{t=\omega ,h}{\Sigma }{\left(1-{\text{exp}}^{-\omega_{t}}\right)}^{\theta }.$$

where


(22) $$\omega_{w}=\frac{\left|w-w^{gt}\right|}{\text{max }\{w,w^{gt}\}},\omega_{h}=\frac{\left|h-h^{gt}\right|}{\text{max }\{h,h^{gt}\}},$$

where *ω* and *h* are the width and height of the predicted bounding box, respectively; $\omega^{gt}$ and $h^{gt}$ are the width and height of the true bounding box, respectively. In summary, the loss function of Inner-SIoU is


(23) $$L_{Inner-SIoU}=1-IoU^{inner}+\frac{\text{Δ}+\text{Ω}}{2},$$

When *α* tends to 0, the angle cost Λ will also tend to 0, which means that the influence of Λ on the Inner-SIoU is greatly reduced. When *α* tends to 3.14*/*4, Λ takes the maximum value, which means that it has the greatest impact on the Inner-SIoU. This approach fully considers the angle between the real bounding box and the predicted bounding box, improving the target box localization ability and prediction accuracy.

## 4 Experimental results and analysis

In this paper, 1,070 images of the longan dataset from the Longan Garden of the Guangdong Academy of Agricultural Sciences are used. After manual screening, annotation, and data expansion, 2,460 longan dataset images are obtained for model training and evaluation. The dataset contains images captured by fruit-picking UAV cameras with different lighting conditions, densities, angles, and longan species, which cover a wide range and have strong generalizability.

**Figure 9:**
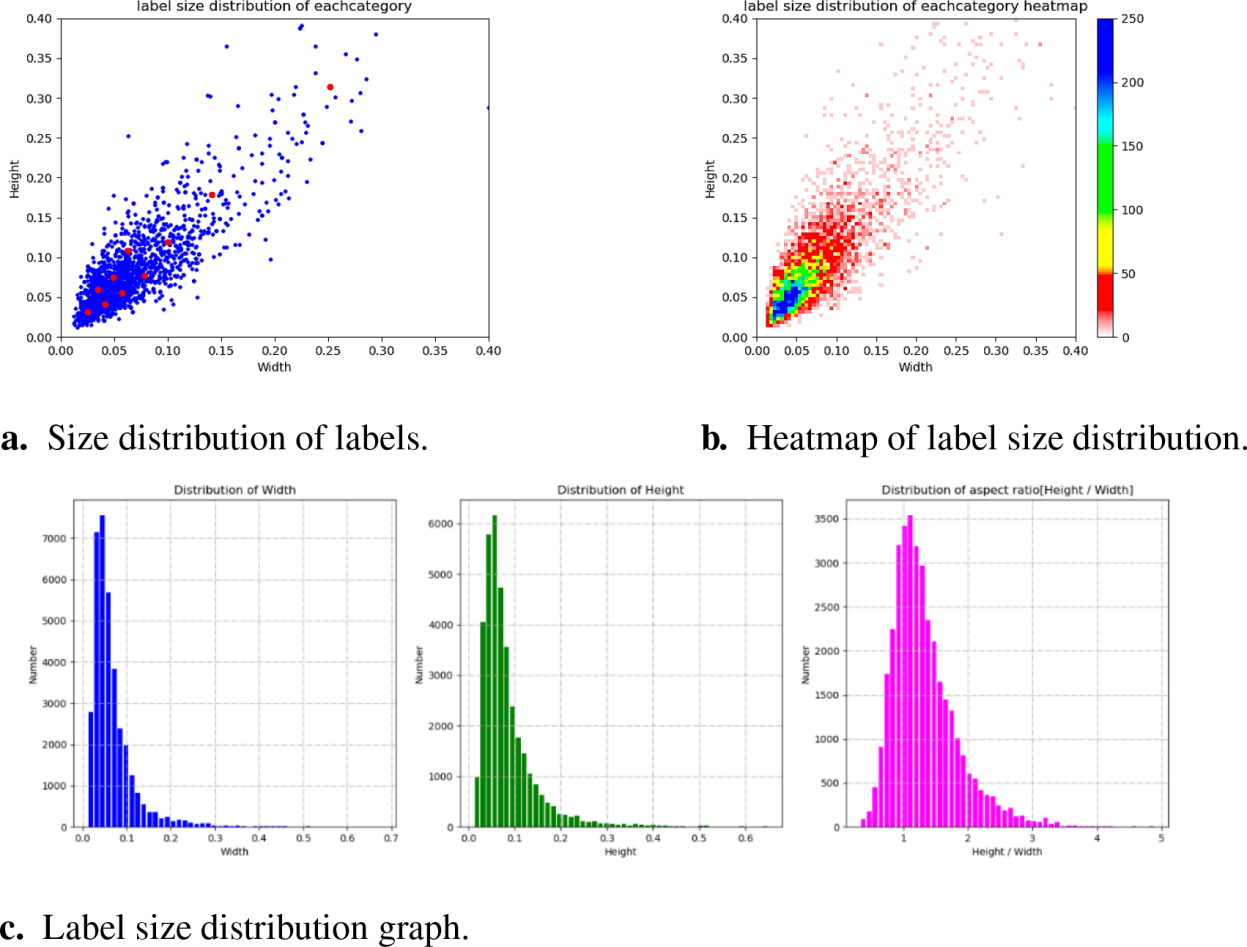
Data size of each category and label size distribution. **(A)** Size distribution of labels. **(B)** Heatmap of label size distribution. **(C)** Label size distribution graph.

This experiment classifies the selected fruit-picking UAV aerial images, of which 1,968 are used for training and 660 are used for testing. The quantity of data and the size distribution of labels for each category in the training set are shown in **Figure 9**. The number of labels in each category varies, and the quantity of data between the corresponding categories varies greatly. In addition, most of the points in the label size distribution map are clustered in the bottom-left corner, while a few points are also clustered in the middle part and the top-right corner. This shows that the longan image dataset contains a large number of small- and partially medium-sized objects with diverse sizes, which is consistent with the background and problems studied in this paper.

The experiment is based on the Ubuntu 18.04 operating system, and the environment is Python3.9, CUDA11.7, and Pytorch2.0. The main specifications are as follows: CPU: AMD EPYC 7402 CPU @ 2.80 GHz; GPU: GPU NVIDIA RTX A4000 16G; RAM: Crucial DDR4 3200 1218G; Mechanical hard disk: WD HC550 16TB; Solid-state drive: SAMSUNG 980 1TB; Motherboard: Supermicro H12SSL.

In order to further validate the robustness of the model and avoid the interference of chance factors on the experiments, this section adopts a fivefold cross-validation method to test the system performance, which randomly and evenly divides the longan string fruit dataset into five subsets. Each experiment uses four of the subsets (1,968 sheets) for training and the remaining one (492 pairs) for testing, and the cross-validation is repeated five times to ensure that each subset is validated once as a test set. To ensure the validity of cross-validation, this experiment ensures that there are no images of the same case between the five subsets when dividing the dataset, i.e., to ensure that there are no overlapping cases between the training set and the test set.

To verify the effectiveness of the proposed model algorithm, we conduct a series of lateral comparison experiments and perform comparative ablation analysis on the corresponding improvement points to verify the advancement of the YOLOv8s-Longan model.

Under the same hyperparameters, the experiments are trained, verified, and tested on the basis of the original model, the training epochs are set to 100, the initial learning rate is 0.01, and the termination learning rate is 1*e*−4.

### 4.1 Experimental evaluation index

To effectively and intuitively demonstrate the improvement effect of YOLOv8 in this paper, the mean average precision (mAP), the number of model parameters (Params), the total floating-point operations (GFLOPs), and the frame rate of refresh [Frames Per Second (FPS)] are used as the evaluation indexes of model performance (Zhou et al., 2024). The evaluation metrics contain precision, deployability, and speed, which are defined as shown in **Table 3**.

**Table 3:** Experimental evaluation indicators.

Indicator Type	Evaluation indexes	Description
Accuracy	mAP@0.5	During the last 10 epochs of model training, the average AP of all images under each category was calculated when the threshold IoU was set to 0.5.
	mAP@0.5–0.95	During the last 10 epochs of model training, the average AP of all images under each category was calculated when the threshold IoU was set to 0.5–0.95.
	Recall	Proportion of positive longan string fruit samples successfully identified by the model.
Deployability	Parameters (m) GFLOPs (G)	The number of parameters in the model. The number of floating-point operations, which measures the computational complexity of the model.
Speed	FPS (img/s)	Refresh frame rate, which indicates how many images are reasoned per second.

Efficient ECA channel attention mechanism, which only adds a small number of parameters but obtains performance gains, has some limitations in dealing with global context dependencies and channel spatial relationships. The DenseCBMA module incorporates the CBAM attention mechanism, adding the spatial attention mechanism on the basis of retaining the original channel attention mechanism, optimizing the network from both the channel and spatial aspects, and improving the feature extraction effect of the model from both the channel and spatial perspectives at the same time. Each DenseNet variant module replaces the first set of Conv-C2f modules in the YOLOv8 backbone network, and the experimental results are shown in **Table 4**.

**Table 4:** Comparison results of the performance of different DenseNet variants.

Variant module	Cross-validation	GFLOPs (G)	Parameters (m)	Recall	mAP@0.5	mAP@0.5–0.95
DenseNet	AVG	35.30	11.10	0.766	81.1%	47.6%
+CA	AVG	+2.70	+0.10	0.754	80.9%	46.4%
+ECA	AVG	+0.12	+0.06	0.759	81.2%	47.1%
+CBMA	AVG	+2.90	+0.10	0.767	81.5%	47.4%
+AMA	AVG	+0.53	+0.08	0.779	82.0%	48.8%

### 4.2 DenseAMA module comparison results

The DenseAMA module is built based on the DenseNet module architecture and introduces the AMA attention mechanism module and the H-wish activation function, which are weighted by average and maximum pooling. The DenseNet, DenseAMA, DenseCA, DenseECA, and DenseCBMA modules are also selected for comparison with other DenseNet variants. The DenseAMA module is proposed on the infrastructure of DenseNet and aims to improve the generalization performance of the classifier through adaptive convolutional kernel tuning while enhancing the flow of information and gradients throughout the network. The DenseCA module introduces the CA attention mechanism in the DenseNet network, which focuses on the attention on the channel dimension, and although it may not be as good as the other 384 attention mechanisms for the case of a small number of channels, it can improve the detection accuracy of the model in scenarios with a large number of channels. The DenseECA module incorporates the efficient ECA channel attention mechanism, which not only adds a small number of parameters but also obtains performance gains, but it has some limitations in dealing with global context dependencies and channel spatial relationships. The DenseCBMA module incorporates the CBAM attention mechanism, adding the spatial attention mechanism on the basis of retaining the original channel attention mechanism, optimizing the network from both the channel and spatial aspects, and improving the feature extraction effect of the model from both the channel and spatial perspectives at the same time. Each DenseNet variant module replaces the first set of Conv-C2f modules in the YOLOv8 backbone network, and the experimental results are shown in **Table 4**.

**Table 4** shows that the DenseAMA module improves the mAP@0.5 by 0.9% compared to the DenseNet module with essentially no change in the number of parameters and the amount of computation. Compared to the rest of the DenseNet variant modules, mAP@0.5 improves by 0.83% on average, thus proving the effectiveness of the DenseAMA module in terms of accuracy.

### 4.3 C2f-Faster-AMA module comparison results

The C2f-Faster-AMA module is built based on the C2f module architecture and introduces the FasterNet and AMA attention mechanism modules. Meanwhile, comparing other residual modules, C2f, C2f-Faster-AMA, C2f-DCNV2, and C2f-DBB residual modules are selected for comparison experiments. The C2f module adopts the concept of multi-level gradient extraction, which enhances the depth of feature extraction and improves the detection accuracy of the model. The C2f-Faster-AMA module is proposed on the basis of FasterNet and aims to reduce the model parameters while maintaining accuracy. The C2f-DCNV2 module adopts a two-branch structure to effectively fuse shared and context-aware weights and aggregate high-frequency local information. The C2f-DBB block aims to improve the feature extraction capability of the network by combining multiple branches for feature extraction using convolutional kernels of different sizes, which are merged or spliced together to form a more master-rich representation. The C2f module is replaced by each residual module in the backbone network, and the experimental results are shown in **Table 5**.

**Table 5:** C2f residual module performance comparison results.

Residual Module	Cross-validation	GFLOPs (G)	Parameters (m)	Recall	mAP@0.5	mAP@0.5–0.95
C2f	AVG	28.4	11.1	0.751	80.4%	46.8%
C2f-DCNV2	AVG	27.1	11.2	0.767	81.2%	47.3%
C2f-DBB	AVG	34.5	13.7	0.759	81.0%	47.2%
C2f-Faster-AMA	AVG	25.6	9.7	0.755	81.8%	47.8%

As shown in **Table 5**, compared to the C2f module, the C2f-Faster-AMA module has 9.8% less computation, 12.7% fewer parameters, and 1.4% improvement in mAP@0.5. Compared to other C2f residual modules, mAP@0.5 improves by 0.67% on average. Thus, the C2f-Faster-AMA module is superior in terms of the number of parameters, the amount of computation, and the prediction accuracy.

### 4.4 Inner-SIoU loss function comparison results

To verify the effectiveness of the loss function Inner-SIoU, the improved Inner-SIoU loss function was compared with Complete Intersection over Union (CIOU), Distance Intersection over Union (DIOU), Extended Intersection over Union (EIOU), and Generalized Intersection over Union (GIOU) in a comparison experiment, and the results are shown in **Table 6**.

**Table 6:** Comparison results of the performance of different loss functions.

Loss function	Cross-validation	Recall	mAP@0.5	mAP@0.5–0.95
CIOU	AVG	0.751	80.4%	46.8%
DIOU	AVG	0.751	81.1%	46.9%
EIOU	AVG	0.773	81.0%	47.6%
GIOU	AVG	0.765	81.1%	47.1%
Inner-SIoU	AVG	0.768	82.1%	47.7%

CIOU, Complete Intersection over Union; DIOU, Distance Intersection over Union; EIOU, Extended Intersection over Union; GIOU, Generalized Intersection over Union.

**Table 6** shows that the model with the Inner-SIoU loss function performs the best, leading the model with the CIOU loss function by 1.7%, which is an average improvement of 1.2% over the other models. In terms of recall, Recall Inner-SIoU still maintains the best recall with an improvement of 2.26% compared to the original model. Under the comprehensive evaluation, the improved loss function is effective, and Inner-SIoU not only improves the detection accuracy but also improves the recall of the model.

### 4.5 Ablation experiment

To analyze the detection performance of the proposed YOLOv8s-Longan algorithm on a dataset of 2,460 UAV aerial longan images, YOLOv8s is the baseline model and does not use pretraining parameters for the models before and after improvement. On the premise of maintaining the same experimental configuration, the detection performance of the proposed YOLOv8s-Longan algorithm improves. The input image resolution is set to the input size of the image taken by the D435i depth camera, which is 848 × 480.

Therefore, an ablation experiment is designed for the UAV aerial longan image dataset, and the experimental parameters are described in Section 4. A comparison of the ablation experimental results of the proposed method is shown in **Table 7**. Model 1 represents the original structure of YOLOv8s, Model 2 represents the integration of the DenseAMA module structure in the front of the YOLOv8s backbone, and Model 3 represents the replacement of the C2f module with the C2f-Faster-AMA module in the back of the YOLOv8s backbone. Model 4 represents the replacement of the original YOLOv8’s neck network with the VOVGSCSPC module of the C2f module, model 5 represents replacing the loss function CIOU in the original YOLOv8s with the improved Inner-SIoU loss function, model 6 represents replacing the backbone overall network structure of the YOLOv8s by combining the DenseAMA module with the C2f-Faster-AMA module, model 7 represents replacing the backbone network of the model 6 with the VOVGSCSPC module to replace the C2f module in the neck network of model 6, and model 8 represents the YOLOv8s-Longan model structure of this paper.

**Table 7:** Comparative results of ablation experiments for YOLOv8s-Longan.

Model	Cross-validation	GFLOPs (G)	Parameters (m)	Recall	mAP@0.5	mAP@0.5–0.95	FPS (img/s)
YOLOv8s	AVG	28.8	11.1	0.751	80.4%	46.8%	115
YOLOv8s + DenseAMA	AVG	35.3	11.1	0.779	82.0%	48.8%	46
YOLOv8s + C2f-Faster-AMA	AVG	25.6	9.7	0.755	81.8%	47.8%	110
YOLOv8s + VOVGSCSPC	AVG	25.2	10.3	0.775	82.0%	47.7%	113
YOLOv8s + Inner-SIoU	AVG	28.8	11.1	0.768	82.1%	47.4%	114
YOLOv8s + DenseAMA + C2f-Faster-AMA	AVG	32.1	9.8	0.780	82.7%	48.4%	43
YOLOv8s + DenseA + C2f-Faster-AMA + VOVGSCSPC	AVG	28.2	8.8	0.778	82.6%	48.3%	45
YOLOv8s-Longan	AVG	28.2	8.8	0.798	84.3%	50.2%	45

According to **Table 7**, integrating the DenseAMA module structure in the front of the YOLOv8s backbone can improve the mAP@0.5 of the model by 1.6%, and replacing the C2f module with the C2FFast-AMA module in the back of the YOLOv8s backbone can improve the mAP@0.5 of the model by 1.4%. Additionally, the combination algorithm of the DenseAMA module and C2F-Fast-AMA module improved the mAP@0.5 of the original YOLOv8s model by 2.3%, thus showing a performance superposition effect. After C2f in the neck network is replaced with the VOVGSCSPC module, the loss function CIOU in the original network structure is changed to the Inner-SIoU loss function to improve global performance. Compared with those of the original YOLOv8s model, the parameters of the proposed YOLOv8s-Longan model are reduced by 20.3%, and the number of calculations in the model is reduced by 2.08%. With the same number of training steps (100 iterations), the recall rate increases by 6.3%, and the prediction accuracy mAP@0.5 increases by 3.9%. It shows that the proposed method not only improves the detection accuracy but also successfully realizes the lightweight nature of the model to meet real-time and accuracy requirements.

### 4.6 Different comparison algorithms

**Figure 10:**
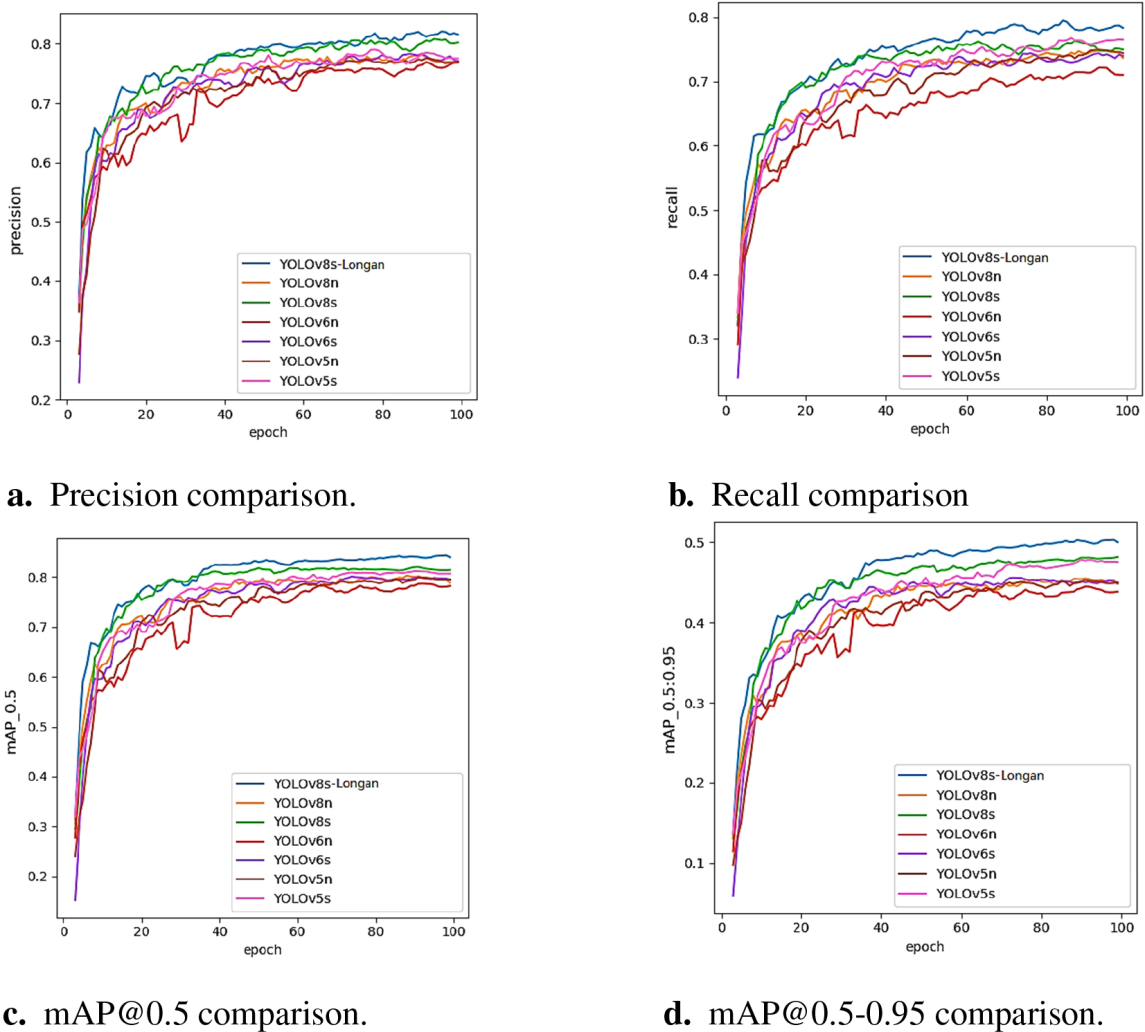
Comparison of object detection algorithms’ indicators. **(A)** Precision comparison. **(B)** Recall comparison. **(C)** mAP@0.5 comparison. **(D)** mAP@0.5-0.95 comparison.

To further verify the efficiency and adaptability of the YOLOv8s-Longan model proposed in this paper for longan string fruit target detection and positioning, the YOLOv8s-Longan model is selected to compare with YOLOv5, YOLOv6, and YOLOv8, which are classic models in the current object detection field. As a mature real-time detection model, YOLOv5 uses Mosaic data enhancement in the input and Focus structure in the Backbone network, which has a good balance between speed and accuracy. YOLOv6 further improves efficiency by introducing RepVGG and EfficientRep modules. As the latest version of the YOLO series, YOLOv8 uses deeper DarkNet-53 as the backbone network and replaces the C3 module in YOLOv5 with the C2f module, which has made significant improvements in lightweight and performance and has strong representability. By comparing the n and s versions of the YOLOv5 and YOLOv8 series proposed by Ultralytics, and the n and s versions of the commonly used YOLOv6 series, six performance indicators are selected. Namely, the amount of computation (GFLOPS), the number of parameters, recall, mAP@0.5 and mAP@0.5–0.95, and FPS are recorded, and the data are shown in **Table 8** and **Figure 10**.

**Table 8:** Comparative experimental results of classical models for object detection.

Model	Cross-validation	GFLOPs (G)	Parameters (m)	Recall	mAP@0.5	mAP@0.5–0.95	FPS (img/s)
YOLOv5n	AVG	7.8	2.65	0.743	79.5%	44.1%	272
YOLOv5s	AVG	24.2	9.15	0.750	79.9%	45.7%	119
YOLOv6n	AVG	13.1	4.5	0.720	78.9%	43.5%	292
YOLOv6s	AVG	44.9	16.4	0.749	79.7%	44.9%	116
YOLOv8n	AVG	8.2	3.0	0.745	79.1%	45.0%	262
YOLOv8s	AVG	28.8	11.1	0.751	80.4%	46.8%	115
YOLOv8s-Longan	AVG	28.8	8.8	0.798	84.3%	50.2%	45

According to the comparative experimental results in **Table 8**, the improved YOLOv8s-Longan model proposed in this paper has higher mAP@0.5 and mAP@0.5–0.95 detection accuracies than other classical models, and the average accuracy of the other mAP@0.5 models increases by 4.72%. The parameters of the improved algorithm in this paper are lower than those of other classical models, the parameters of the YOLOv8s-Longan model are only half of those of the YOLOv6s model, and the parameters of the YOLOv8s-Longan model are reduced by 23.6% on average compared with those of other models with the same specifications. From the perspective of various indicators, the improved model algorithm in this paper has the best comprehensive performance and has good detection ability for longan string fruit images. This model not only improves detection accuracy but is also lightweight and can meet real-time and accurate requirements, demonstrating the obvious superiority of the YOLOv8s-Longan target detection model.

To better show the effectiveness of the improved algorithm, various classical accuracy detection models and the YOLOv8s-Longan model in the training process are compared with the changes in four indicators: accuracy, recall rate, mAP@0.5, and mAP@0.5–0.95. The experimental results are shown in **Figure 10**. With an increase in the number of iterations, all the comparison algorithms can finally reach convergence, but the four indicators of the improved YOLOv8s-Longan model are significantly greater than those of all the classical detection models. A comparison of the mAP@0.5 and mAP@0.5–0.95 curves is shown in **Figures 10C, D**. The mAP of the improved algorithm is greater than that of the original YOLOv8s benchmark model when training for 100 rounds, which proves that the YOLOv8s-Longan model in this paper can effectively improve the ability to detect longan bunk fruit compared with the original benchmark model.

**Figure 11:**
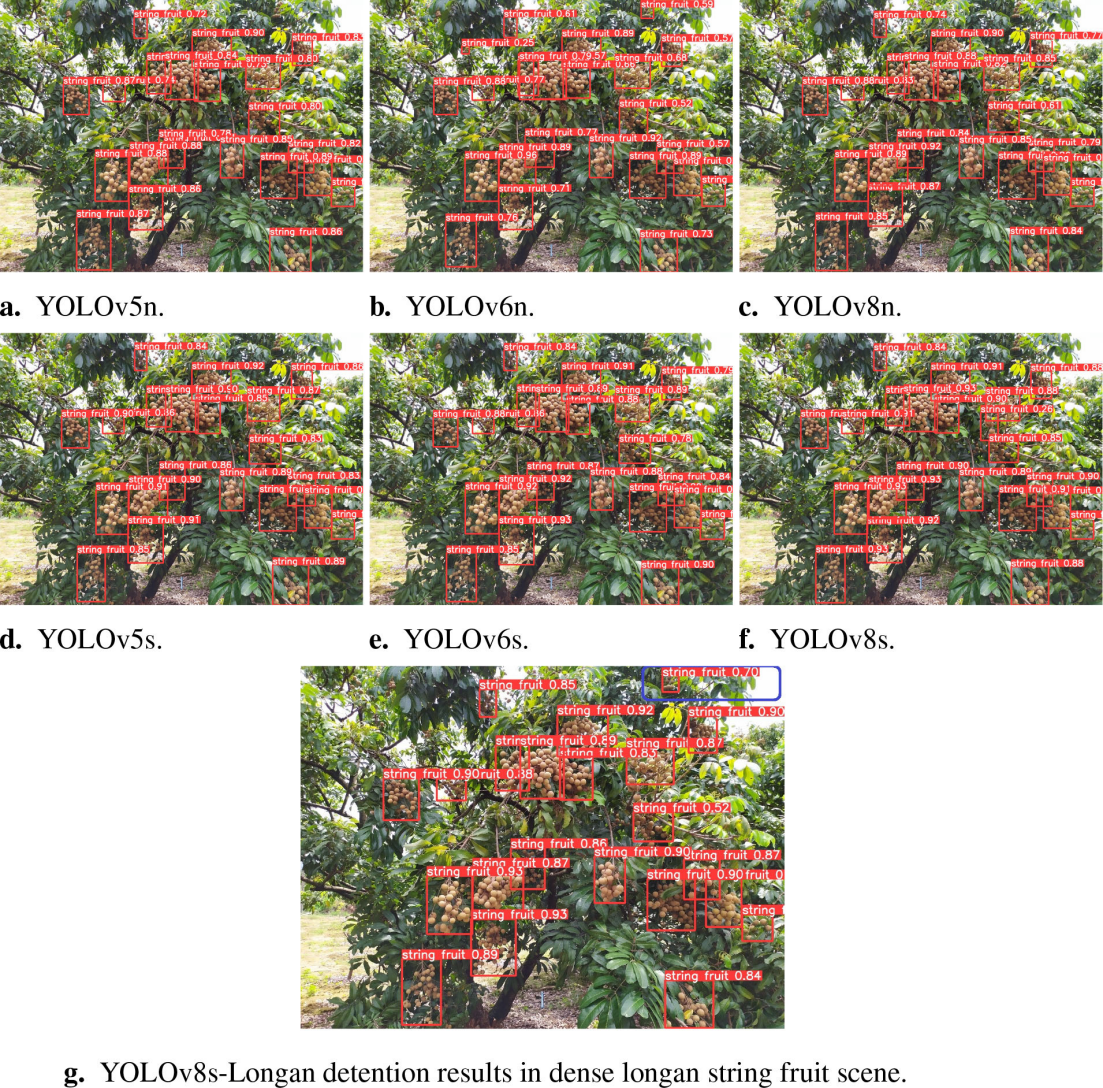
Prediction comparison of different network models for identification. **(A)** YOLOv5n. **(B)** YOLOv6n. **(C)** YOLOv8n. **(D)** YOLOv8n. **(E)** YOLOv6s. **(F)** YOLOv8s. **(G)** YOLOv8s-Longan detention results in dense longan string fruit scene.

The Longan Garden of the Guangdong Academy of Agricultural Sciences was used to test some of the 1,070 longan dataset images to evaluate the effect before and after the improvement more intuitively. Comparing **Figure 11** shows that except for YOLOv6s in **Figure 11D** and YOLOv8s-Longan in **Figure 11G**, there is no missing detection in the upper-right corner of the dense longan string fruit scene; other detection models fail to detect longan string fruit in the upper-right corner of the figure. Additionally, compared with **Figures 11D, G**, the overall accuracy of the YOLOv8s-Longan model in image detection is much greater than that of the YOLOv6s model, and the prediction accuracy of the YOLOv6s model increases by 21.1% on average. The improved model has higher detection accuracy, and the detection performance is significantly improved.

**Figure 12:**
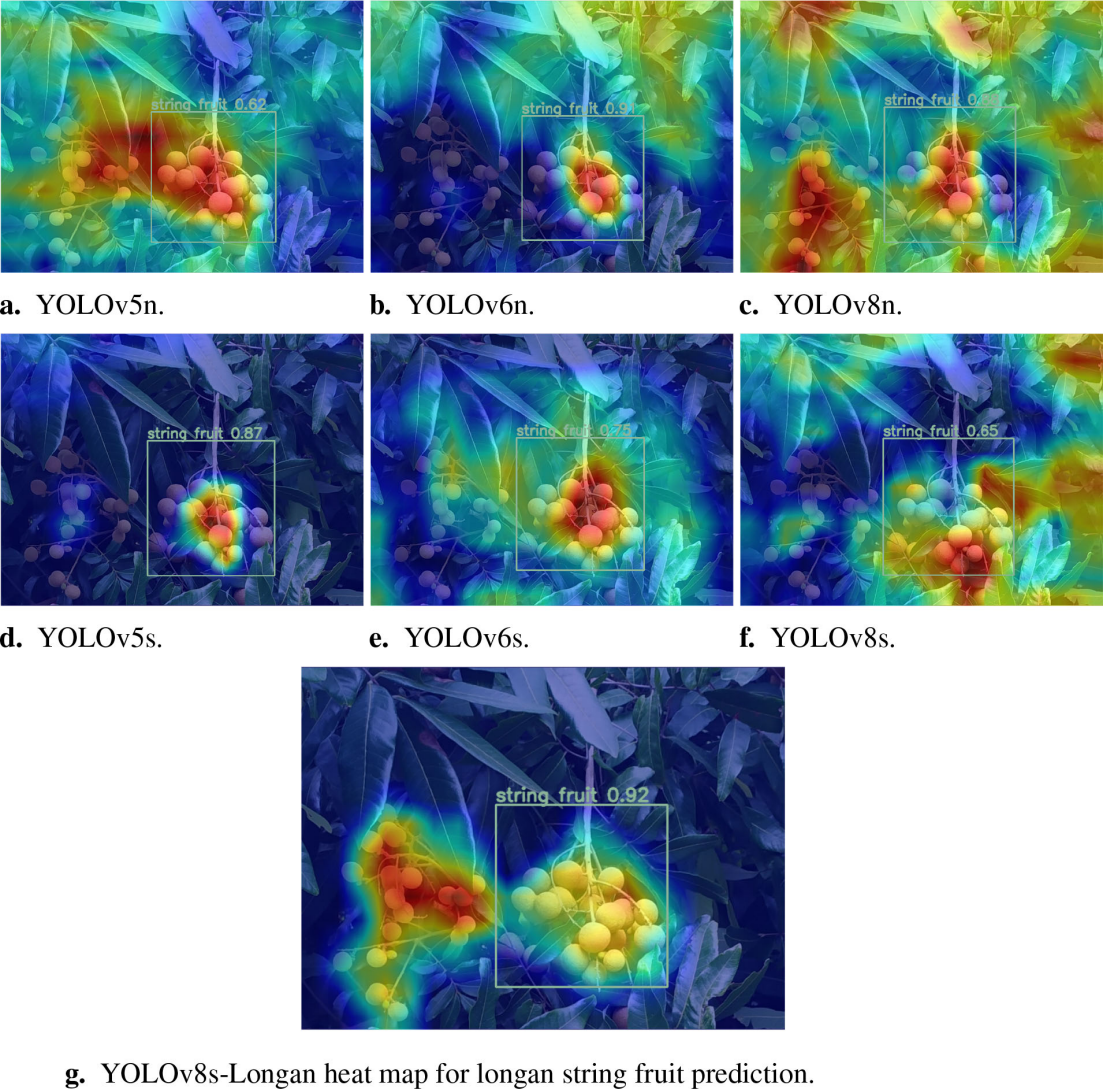
Comparison of heatmaps for prediction of longan fruit recognition by different network models. **(A)** YOLOv5n. **(B)** YOLOv6n. **(C)** YOLOv8n. **(D)** YOLOv8n. **(E)** YOLOv6s. **(F)** YOLOv8s. **(G)** YOLOv8s-Longan heat map for longan string fruit prediction.

To further explore the improvement of the YOLOv8s-Longan model algorithm, a heatmap visualization comparison and analysis of the detection effect are performed, and the specific results are shown in **Figure 12**.

Specifically, **Figures 12A, D** show the heatmap visualization comparison of the YOLOv5 model, in which **Figure 12A** only vaguely identifies the approximate position of the longan string fruit, and **Figure 12D** only identifies the center position of the right longan string fruit but does not identify the left longan string fruit. **Figures 12B, E** show the heatmap visual comparison of the YOLOv6 model. This group of figures can only identify the center position of the right longan string fruit and has obvious false detection of the surrounding green leaf environment. A heatmap of the YOLOv8 model is shown in **Figures 12C, F**. In **Figure 12C**, the approximate position of the longan string fruit on the left and right sides is fuzzy, but the surrounding green leaves are clearly misidentified. **Figure 12F** shows the approximate identification of the peripheral outline of the longan string fruit on the right. **Figure 12G** shows a heatmap visual comparison between the YOLOv8s-Longan model and other classical detection models. The improved YOLOv8s-Longan model can perfectly identify the irregular peripheral contour of longan string fruit, and there is no false detection of the surrounding green leaves or other interference objects. Therefore, the YOLOv8s-Longan model performs well in improving object detection accuracy and solving the problems of missed and false detections, which significantly improves the object detection task.

### 4.7 Detection effects in different natural scenes

**Figure 13:**
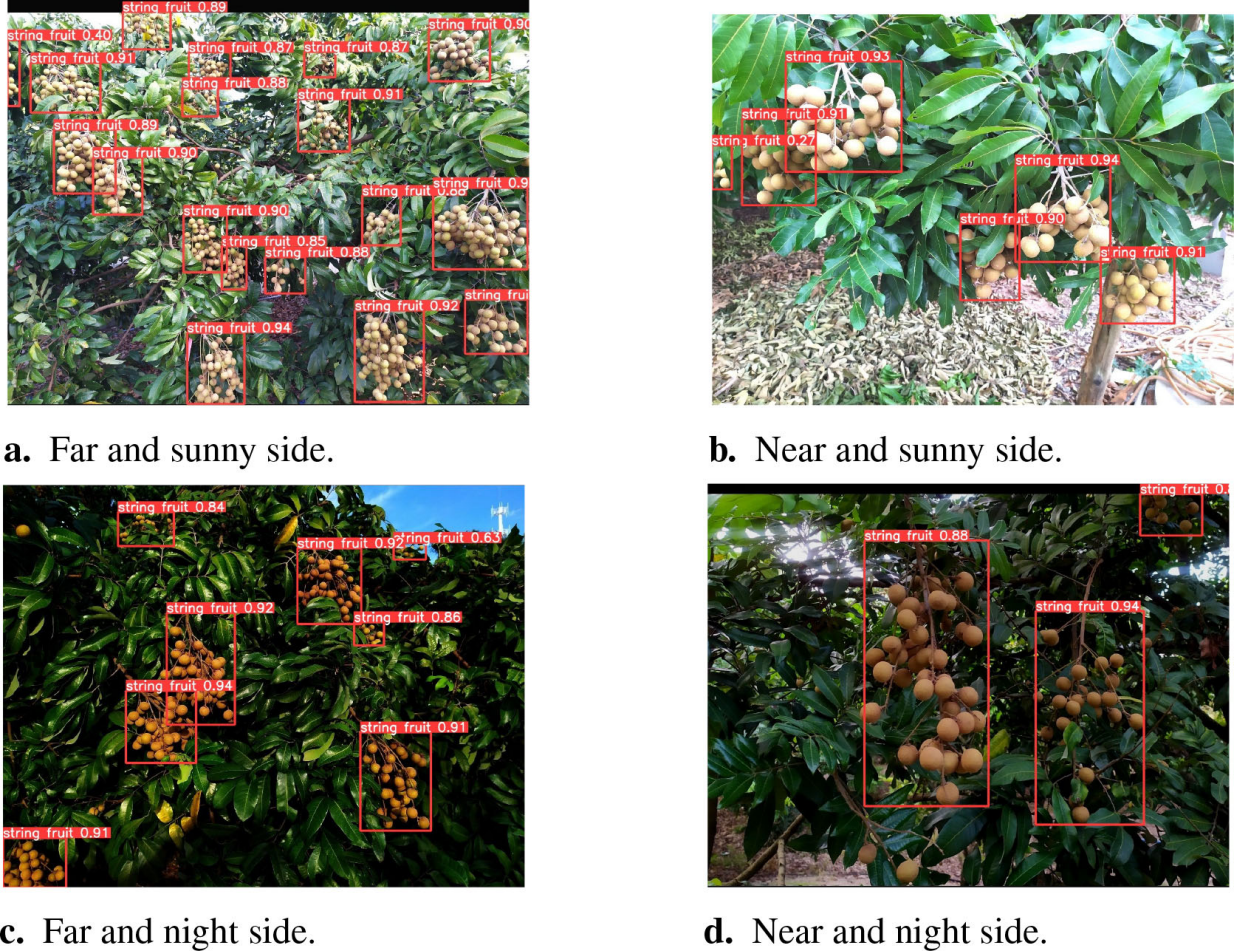
Comparison of detection results under different environmental conditions. **(A)** Far and sunny side. **(B)** Near and sunny side. **(C)** Far and night side. **(D)** Near and night side.

In this section, the performance of the YOLOv8s-Longan model under different lighting conditions is evaluated in detail. In the frontal illumination environment, **Figures 13A, B** demonstrate that the model can accurately identify the target at different distances, far and near. **Figures 13C, D** are in the backlight condition; the model is still able to accurately identify the longan string fruit without being affected by the light intensity. As shown in **Figures 13A, C**, the recognition of longan strings in long-distance scenes proves that the model can maintain accurate detection of longan string fruit regardless of the lighting environment or scene distance. Comprehensive **Figure 13** shows that the YOLOv8s-Longan model shows strong robustness regardless of the changes in lighting conditions or near and far scenes and successfully realizes the accurate detection of targets under different environmental conditions.

**Figure 14:**
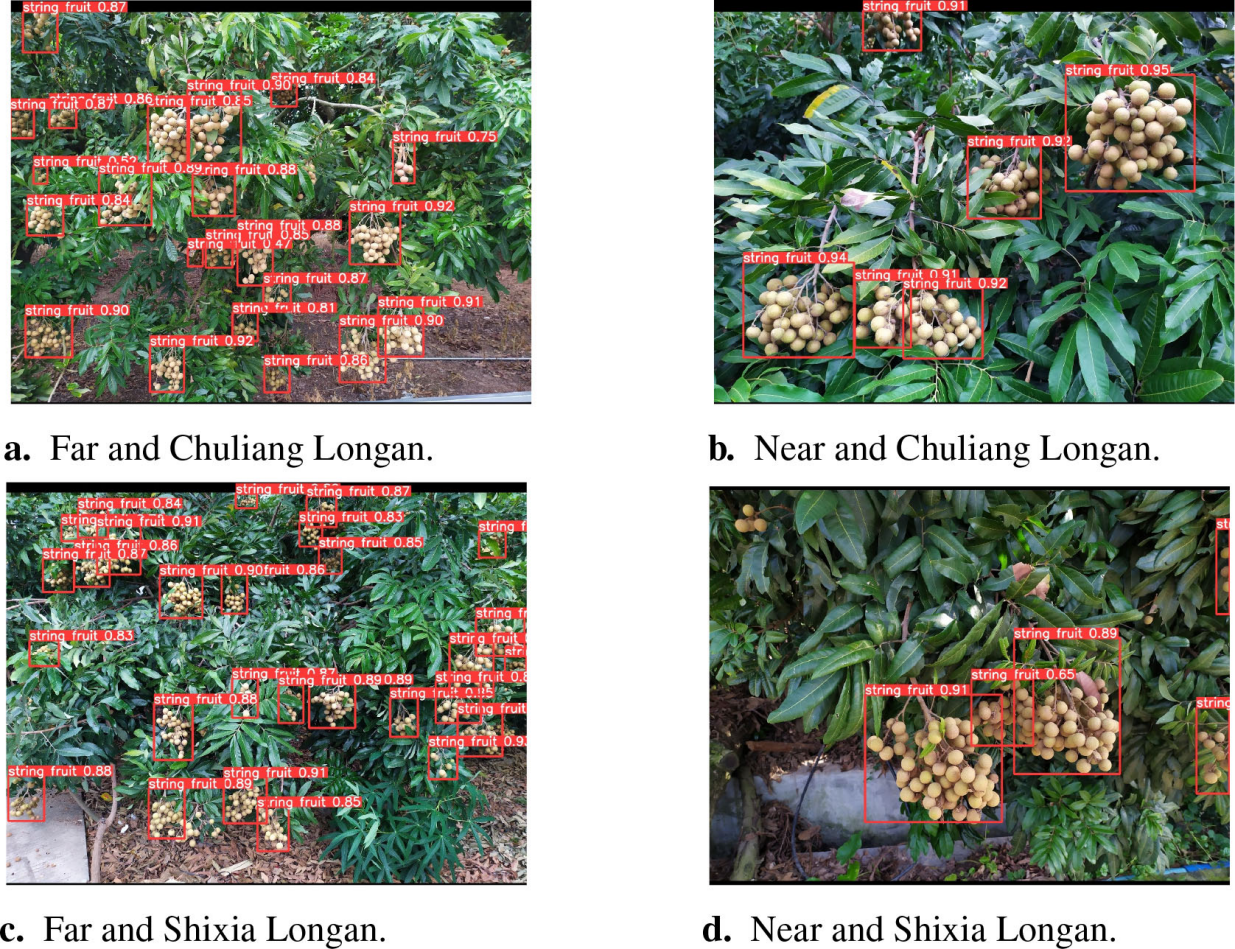
Comparison of the detection results for different longan varieties. **(A)** Far and Chuliang Longan. **(B)** Near and Chuliang Longan. **(C)** Far and Shixia Longan. **(D)** Near and Shixia Longan.

In order to verify the robustness of the YOLOv8s-Longan model for the recognition of different longan varieties, especially the detection ability of the model for different longan varieties in the same environment, **Figures 14A, B** show the detection results for the Chuliang longan, while **Figures 14C, D** show the detection results for the Shixia longan. There were obvious differences in the color, size, and shape of the two longan fruits, and different appearance characteristics were reflected at different distances and light conditions. Based on **Figure 14**, it can be seen that the model can accurately identify longan string fruit, which indicates that the YOLOv8s-Longan model shows excellent generalization performance in identifying different longan varieties.

### 4.8 The real-time deployment test

**Figure 15:**
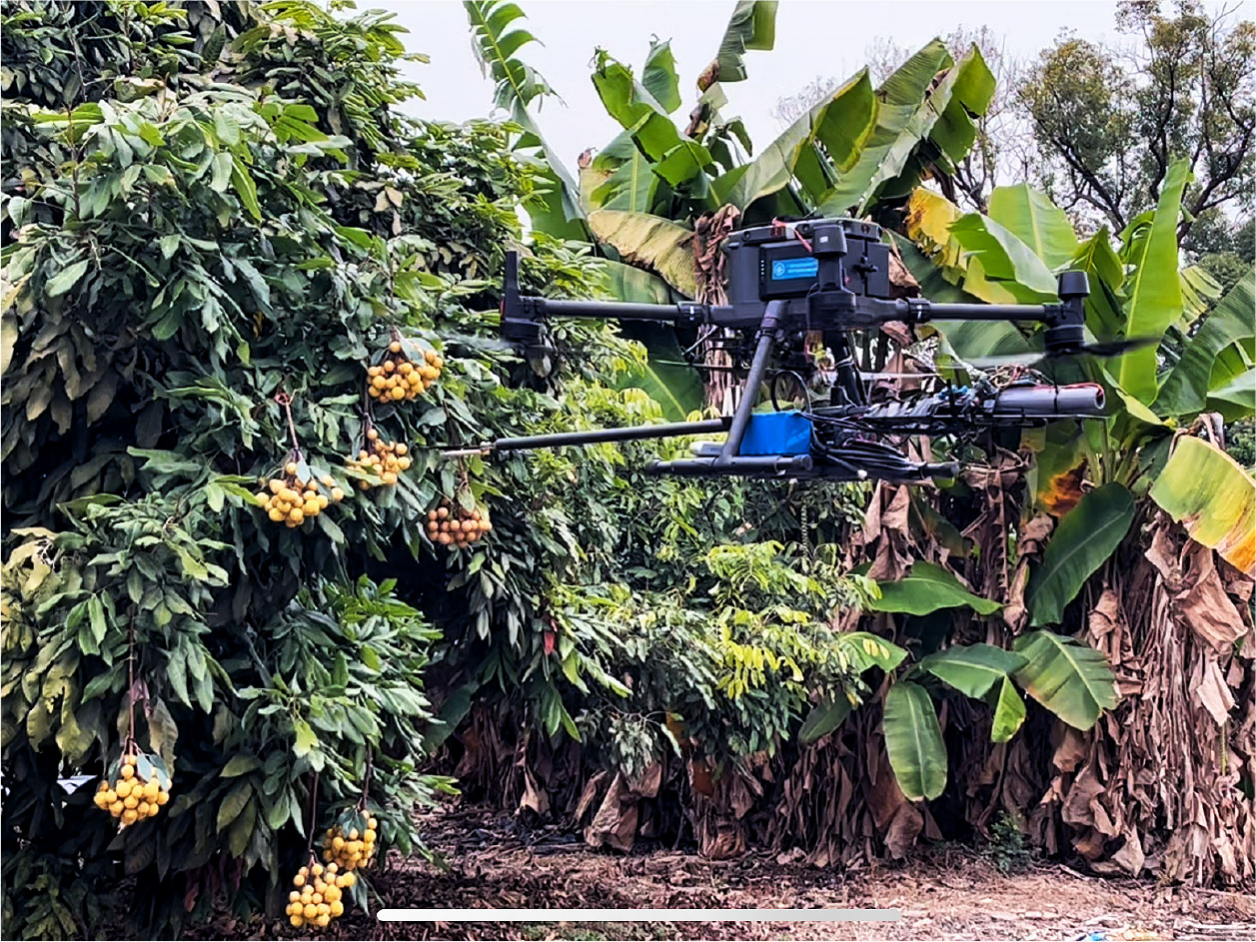
The UAV test scenario. UAV, unmanned aerial vehicle.

To verify the practical deployment capability of the proposed YOLOv8s-Longan model for the UAV, in this experiment, DJI M300 RTK model UAV and Intel RealSense D435i camera are selected, and the YOLOv8s-Longan model is deployed to the NanoPi-R5C-Combo onboard computer. The performance of string fruit recognition is tested on Chuliang and Shixia longan scenes in the longan garden of the Guangdong Academy of Agricultural Sciences. The test scenario is shown in **Figure 15**, and the recognition results are shown in **Table 9**.

**Table 9:** Recognition results of YOLOv8s-Longan model in different natural scenes.

Different natural scenes	Number of true longan string clusters	Identify the correct number of longan string clusters	Identifying the wrong number of longan string clusters	Accuracy
Close, sunny side	7	7	0	100%
Close, night side	9	8	1	88.9%
Far, sunny side	15	13	2	86.7%
Far, night side	17	14	3	82.3%

During the actual test, the NanoPi-R5C-Combo on-board computer deploys the lightweight model with a parameter count of 8.83M with 18.1 MB of memory, and the model can process 45 to 50 images per second, which can meet the real-time recognition of longan string in real-time by the UAV. From **Table 9**, the YOLOv8s-Longan model has good recognition and detection results for both Chuliang and Shixia longan varieties in different natural scenarios. Among the 48 clusters of identified longan string, 42 clusters of string are accurately identified, and the average recognition accuracy of the YOLOv8s-Longan model is 87.5%, which can satisfy the need of the UAV for lightweight and accurate recognition of longan string. Among the six clusters of longan string that were missed, four clusters of string are occluded by the transition of longan string in front of them and thus identified as one cluster of longan string by the model; the other two clusters of string are missed because they are located inside the center of the fruit tree under cloudy conditions, which prevented them from being accurately identified by the model.

## 5 Conclusion

In this paper, a fast and accurate detection scheme based on deep learning is proposed for the UAV aerial longan image dataset. First, the Intel RealSense D435i depth camera is mounted on the fruit-picking UAV to collect longan string fruit data. Second, in order to reduce the computing requirements and memory usage of airborne computing equipment and improve the fast and accurate detection accuracy of longan string fruit, the YOLOv8s-Longan deep learning model is proposed.

The experimental results show that the recall and mAP@0.5 of the improved model proposed in this paper increase by 6.3% and 3.9%, respectively, on the longan string fruit dataset, and the parameter quantity of the improved model decreases by 20.3%. Compared with the other three YOLO series classical algorithms, the improved model algorithm in this paper is feasible, which improves the detection accuracy of longan string fruit targets and greatly reduces the number of missed and false detections of occluded targets.

In the future, the training speed of the model and the ability of the object detection model to resist environmental interference will be further improved, and the robustness, generalization ability, and application prospect of the model will be enhanced. In future work, we will analyze the maturity and disease and insect pests of longan string fruit through the model and provide customized picking strategies, which will help to improve the yield and quality of longan, promote the income growth of fruit farmers, and promote the sustainable development of longan cultivation industry.

## Data Availability

The raw data supporting the conclusions of this article will be made available by the authors, without undue reservation.
